# Fluid Candidate Biomarkers for Alzheimer’s Disease: A Precision Medicine Approach

**DOI:** 10.3390/jpm10040221

**Published:** 2020-11-11

**Authors:** Eleonora Del Prete, Maria Francesca Beatino, Nicole Campese, Linda Giampietri, Gabriele Siciliano, Roberto Ceravolo, Filippo Baldacci

**Affiliations:** Neurology Unit, Clinical and Experimental Medicine Department, University of Pisa, 56126 Pisa, Italy; mariaf.beatino@gmail.com (M.F.B.); campesenicole@gmail.com (N.C.); lindagiampietri@gmail.com (L.G.); gabriele.siciliano@unipi.it (G.S.); r.ceravolo@med.unipi.it (R.C.); filippo.baldacci@unipi.it (F.B.)

**Keywords:** biomarkers, Alzheimer’s disease, neurodegeneration, cerebrospinal fluid, mild cognitive impairment, synaptic biomarkers, neuroinflammation, neurofilament light chain

## Abstract

A plethora of dynamic pathophysiological mechanisms underpins highly heterogeneous phenotypes in the field of dementia, particularly in Alzheimer’s disease (AD). In such a faceted scenario, a biomarker-guided approach, through the implementation of specific fluid biomarkers individually reflecting distinct molecular pathways in the brain, may help establish a proper clinical diagnosis, even in its preclinical stages. Recently, ultrasensitive assays may detect different neurodegenerative mechanisms in blood earlier. ß-amyloid (Aß) peptides, phosphorylated-tau (p-tau), and neurofilament light chain (NFL) measured in blood are gaining momentum as candidate biomarkers for AD. P-tau is currently the more convincing plasma biomarker for the diagnostic workup of AD. The clinical role of plasma Aβ peptides should be better elucidated with further studies that also compare the accuracy of the different ultrasensitive techniques. Blood NFL is promising as a proxy of neurodegeneration process *tout court*. Protein misfolding amplification assays can accurately detect α-synuclein in cerebrospinal fluid (CSF), thus representing advancement in the pathologic stratification of AD. In CSF, neurogranin and YKL-40 are further candidate biomarkers tracking synaptic disruption and neuroinflammation, which are additional key pathophysiological pathways related to AD genesis. Advanced statistical analysis using clinical scores and biomarker data to bring together individuals with AD from large heterogeneous cohorts into consistent clusters may promote the discovery of pathophysiological causes and detection of tailored treatments.

## 1. Introduction

Alzheimer’s disease (AD) is the most common neurodegenerative disease (NDD), with 5.8 million Americans aged 65 years and older living with AD in 2020 [1]. Since Alois Alzheimer’s first description of the typical histological alterations of neuritic plaques (NP) and neurofibrillary tangles (NFT) in 1906 [2], more than eighty years have passed before amyloid beta (Aβ) and phosphorylated-tau (p-tau) were identified as the main component of NP and NFT, respectively [3]. In 1984, the National Institute of Neurological and Communicative Disorders and Stroke-Alzheimer’s Disease and Related Disorders Association (NINCDS-ADRDA) [4] set postmortem examination as the reference standard of AD diagnosis. Since then, the broad phenotypical variability of neurodegenerative diseases (NDDs) has pushed the efforts toward developing a classification based on the main misfolded protein deposition [5,6]. Nevertheless, the occurrence of these aggregates in multiple combinations is frequent, and NDDs are rather emerging as a spectrum of disorders characterized by the loss of proteostasis [7]. 

**Figure 1:**
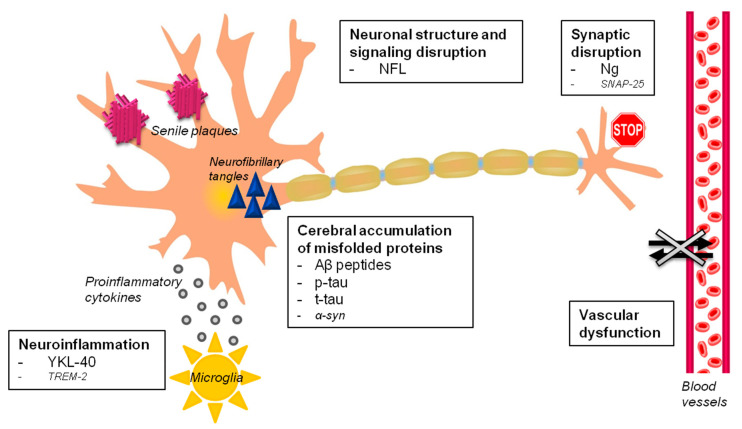
Alzheimer’s disease fluid biomarkers. The major pathophysiological processes involved in Alzheimer’s disease (in bold) with validated and proposed fluid biomarkers are schematically represented. Fluid biomarkers of vascular dysfunction, and of TAR DNA binding protein 43 (TDP-43) and α-syn pathologies are still missing. Abbreviations: *Aβ*, β-amyloid, *α-syn*, α-synuclein; *NFL*, neurofilament light chain; *Ng*, neurogranin; *p-tau*, phosphorylated tau protein; *t-tau*, total tau protein, synaptosomal-associated protein 25 (SNAP-25), and triggering receptor expressed on myeloid cells 2 (TREM2).

Due to the failure of numerous trials against amyloid pathology, the idea of “one drug fits all” treatment as an ultimate solution for an AD cure is fading [8]. In brief, the current framework on AD is more complex than previously thought because AD is not a mere plaque and tangle disorder. The following pathophysiological pathways leading to neurodegeneration have been recognized as clearly implicated in AD pathogenesis: (1) accumulation of misfolded proteins in the brain (Aβ peptides, tau and p-tau proteins, other co-pathologies), (2) vascular dysfunction, (3) synaptic disruption, and (4) neuroinflammation. The discovery of biomarkers indicating the modification of these processes at different levels in space and time is gaining momentum, especially in design tailored disease-modifying trials (Figure 1). 

Our aim is to review the development of novel candidate fluid biomarkers tracking these key pathophysiological mechanisms in different matrices, especially cerebrospinal fluid (CSF) and blood. In relation to AD, we mainly focused on the diagnostic and prognostic value of these biomarkers, with particular attention to the novel ultrasensitive techniques.

## 2. Literature Search Methods

**Figure 2:**
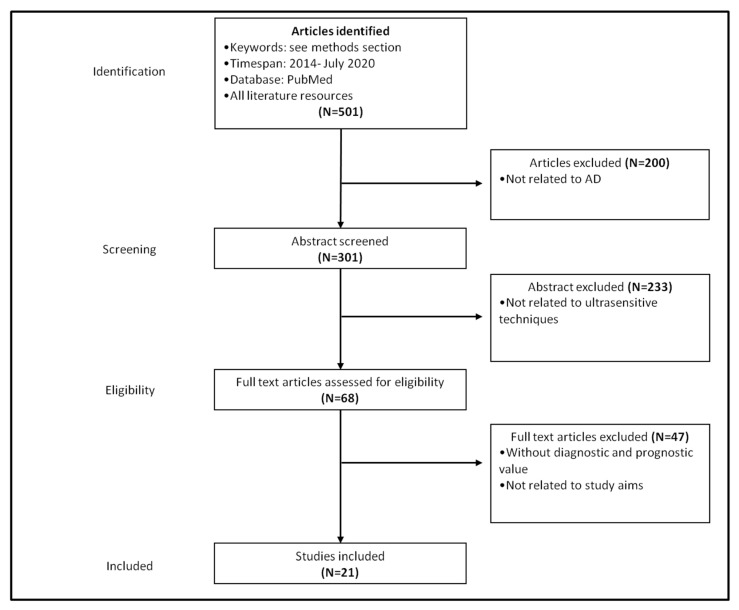
Flowchart displaying the article selection process.

We performed a narrative review of literature focusing on novel candidate fluid biomarkers for AD. A systematic review of literature focused on plasma biomarkers detected by means of novel ultrasensitive techniques was performed in PubMed. We used the combination of the keywords “plasma”, “serum”, “amyloid-β”, “NFL” (neurofilament light chain), “p-tau”, “p-tau181”, “phopsho-tau181”, “phosphorylated tau181”, “t-tau”, “Simoa”, “immunoassay”, “immunomagnetic reduction”, “fully automated”, “immuno-infrared sensor”, “mass spectrometry”, and “multimer detection system”. Only papers in English published between 2014 and July 2020 and focused on AD were included in the final analysis. Overall, we identified 21 studies that provided relevant diagnostic and/or prognostic information (Figure 2). Among them, 10 were focused on amyloid-β peptides, 7 were focused on p-tau or tau or both, and 4 were focused on NFL. For each paper, the study population, the study design (cross-sectional, perspective, retrospective), and the diagnostic and/or prognostic value of the investigated biomarker were analyzed. We classified the diagnostic value of each biomarker according to previously published recommendations as follows: “excellent” (area under ROC curve [AuROC] 0.90–1.00), “good” (AuROC 0.80–0.89), “fair or moderate” (AuROC 0.70–0.79), “poor” (AuROC 0.60–0.69), or “fail or insufficient” (i.e., no discriminatory capacity) (AuROC 0.50–0.59).

## 3. Toward a Pathophysiological Definition of Alzheimer’s Disease

With the 1984 NINCDS-ADRDA criteria, the accuracy for probable AD diagnosis was suboptimal, with sensitivity between 70.9% and 87.3% and specificity between 44.3% and 70.8% [9]. The definite diagnosis relied on postmortem examination, with obvious limitations, since it is not applicable in vivo. For this reason, the International Working Group (IWG) [10] and later the National Institute on Aging and Alzheimer’s Association (NIA-AA) [11] published novel criteria for the diagnosis of AD incorporating in vivo biomarkers. According to the 2007 IWG criteria, AD can be identified in vivo by the presence of amnestic syndrome of the hippocampal type, which is characterized by low free recall that does not improve with cueing. Moreover, biomarkers must be consistent with AD pathology. These biomarkers are pathophysiological and topographical. The pathophysiological ones are low CSF Aβ1-42 peptide concentration, high CSF total tau (t-tau) or p-tau levels, and an increased cerebral uptake of amyloid tracers (e.g., Pittsburgh compound) with PET. The formers are hippocampal atrophy on volumetric Magnetic Resonance Imnaging (MRI) and cortical regional hypometabolism on fluorodeoxyglucose FDG-PET, involving bilateral temporal parietal regions and posterior cingulate. IWG criteria managed to move from the static and binary/dichotomic vision of AD as a clinicopathological entity to its current dynamic clinical-radio-biological description [10]. The subsequent 2010 revision of IWG criteria overtook the amnestic-centered concept of AD and broadened the spectrum, adding the rarer atypical forms of AD, such as primary progressive non-fluent aphasia, in particular logopenic aphasia, posterior cortical atrophy, and frontal variant AD. The-so-called “asymptomatic at risk of AD” condition without clinical symptoms but with positive biomarkers of AD pathology was stated out, as well as the concept of “mixed AD”, implying the co-occurrence of clinical and biological features of other disease, such as parkinsonism (e.g., Lewy body pathology) or cerebrovascular disease [12]. Later on, these concepts were implemented in the IWG-2 criteria (2014) [13], where clinical diagnosis required specifying whether typical or atypical AD phenotypes occurred. Furthermore, the condition of a preclinical AD stage (for asymptomatic at risk and presymptomatic subjects) was defined in the presence of cognitive normal individuals with biomarkers indicative of AD pathophysiological process. Topographical biomarkers were used only for disease staging and monitoring. In parallel with IWG criteria, the NIA-AA diagnostic guidelines developed in 2011 [14,15,16] moved forward, defining the concept of mild cognitive impairment (MCI) due to AD (clinical MCI individuals with biomarkers indicating AD pathology). In fact, MCI due to AD had a high likelihood of developing AD over time. Subsequently, in 2016, the joint IWG-Alzheimer’s Association (IWG-AA) formalized a purely biological definition of AD, based on the positivity of biomarkers of both amyloidosis and tauopathy [17]. In the same years, the “A/T/N” classification system for AD was published. In this classification, the validated AD biomarkers were reported into three binary categories (presence or absence) based on the nature of the pathophysiology. “A” refers to the ß-amyloid pathology (cerebral amyloid PET or CSF Aß42); “T,” refers to taupathology (CSF p-tau, or cerebral tau PET); and “N” refers to neurodegeneration or neuronal injury *tout court* ([18F]-fluorodeoxyglucose-PET, structural MRI, or CSF total tau [18]). This unbiased biomarker-based scheme was recently incorporated in the current NIA-AA criteria published in 2018, with the addition of C for clinical change, to integrate the biomarkers condition with clinical cognitive status [19]. All A+ individuals are considered part of the “Alzheimer’s continuum”, while only A+ and T+ define AD. Non AD-specific parameters, namely neurodegenerative/neuronal injury biomarkers (N) and cognitive symptoms (C), define staging [19]. A- individuals fall either in the “normal AD biomarker” category with A-T-(N-), or “Suspected non-Alzheimer’s pathophysiology” (SNAP) with A-T+(N)-, A-T-(N)+, or A-T+(N)+. 

Among the mimics of typical AD-type dementia, Primary Age-Related Tauopathy (PART) should be mentioned [20,21]. PART identifies individuals with cerebral NFT indistinguishable from those of AD, in the absence of Aβ plaques; notably, NFT are restricted to the medial temporal lobe, basal forebrain, brainstem, and olfactory areas [21]. At a clinical level, associated manifestations range from normal cognition to amnesic cognitive impairment, but they are rarely a frank dementia. Similarly, a recently described entity is the limbic-predominant age-related TDP-43 encephalopathy (LATE) [22]. LATE is a common TDP-43 proteinopathy that generally affects older adults, and it is frequently associated with hippocampal sclerosis. Aβ plaques or tauopathy may also coexist. Generally, co-pathologies in AD subjects are common with approximately 30% of AD patients showing a cerebrovascular disease [23]. The concomitant deposition of Aβ and α-syn proteins is also described in postmortem examination in about 30% of AD individuals [24,25] but also in up to 40% of patients with Parkinson’s disease (PD), Parkinson disease dementia (PDD), and dementia with Lewy bodies (DLB) clinical diagnoses [26,27,28].

Thus, in this scenario, it is likely that no single biomarker could reach a 100% diagnostic accuracy, being AD biologically multifaceted with a clinical picture reflecting pathology only in terms of probabilistic association. Despite these intrinsic limitations, the use of core biomarkers in the AD diagnostic workup improves accuracy (up to 90%) with a relevant impact on AD stratification and selection for disease-modifying trials tailored against Aß and tau pathologies [29]. 

To date, the neuropathological hallmarks of AD remain extracellular Aβ plaques and NFTs [30,31]. First proposed in 1992, the “amyloid cascade hypothesis” [32] has been later corroborated by genetic and biochemical data and currently represents the dominant pathogenetic model of AD. According to this hypothesis, the deposition of fibrillar Aβ plaques within the brain promotes the accumulation of NFTs, synaptic disintegration and neuronal death by inflammatory mechanisms, modification of ions homeostasis, kinase/phosphatase activity, and oxidative stress [33]. In particular, Aβ plaques create an unique environment that facilitates tau aggregation, initially as dystrophic neurites surrounding Aβ plaques, followed by the formation and spread throughout the brain in a prion-like manner of NFTs and neuropil threads [34]. NFTs are characteristic of AD and are composed of hyperphosphorylated tau [35,36,37]. The hyperphosphorylation of tau protein reduces its affinity for microtubules and promotes its capacity to aggregate and fibrillize [38]. Therefore, microtubules are destabilized, and axonal transport is impaired [39]. 

The hyperphosphorylated tau could also migrate in the somatodendridic compartments where it interacts with Aβ and enhances synaptotoxicity [40], finally causing cell death due to a toxic gain of function mechanism [41,42]. 

At the same time, much interest is growing around the role of inflammation in the pathogenesis of AD. The contribution of inflammation to the pathophysiology of AD has been already hypothesized more than 20 years ago [43,44,45]. The attention has been focused especially on microglia activation, which seems to occur decades before AD onset [46,47,48]. Furthermore, a correlation between neuroinflammation and amyloid or tau accumulation in the human brain has been reported in several investigations [46,49,50,51]. The microglial activation produces two different phenotypes. The microglial “pro-inflammatory” phenotype (M1) displays pro-inflammatory cytokines (IL-1β, IL-6, IL-12, tumor necrosis factor (TNF)-α, CCL2), nitric oxide, reactive oxygen and nitrogen species. The “anti-inflammatory” one (M2) sustains the production of IL-10 and TGF-β, and it increases the expression of neurotrophic factors (nerve-derived growth factor (NGF), brain-derived neurotrophic factor (BDNF), neurotrophins, glial cell–derived neurotrophic factor (GDNF)), and several other signals involved in downregulation, protection, or repair processes [52]. The chronic stimulus on microglia by Aβ peptides accumulation is likely to lead to a protracted inflammation, and, in turn, increase Aβ deposition, in a vicious circle [53]. The inflammatory state would promote the production and release of pro-inflammatory cytokines, which could themselves have a detrimental effect by inducing neuronal cell death.

Another relevant key pathophysiological mechanism that contributes to AD is vascular dysregulation [54]. Several pieces of evidence support the role of chronic cerebral hypoperfusion as the *primum movens* of AD pathology [55,56]. Hypoxia can activate β-secretase-1 and γ-secretase as well as increase Aβ peptides accumulation [57]. Furthermore, the reduced supply of oxygen and nutrients affects neurons per se, and, in turn, it promotes blood–brain barrier dysfunction, increasing oxidative stress and inflammation [58]. Since Aβ deposition derives essentially from an imbalance between production and removal, clearing system impairment is emerging as a further key pathophysiological mechanism leading to AD. In particular, this mechanism involves the alteration of astroglial-mediated interstitial fluid (ISF) bulk flow or glymphatic system [59,60]. This pathway is mainly modulated by the sleep–wake cycle and seems to be important for the sleep-driven clearance of Aβ [61]. Vascular pathology seems to be additive or even synergic to AD pathology as a cause of cognitive impairment [62,63]. This cross-talk is most evident for cerebral amyloid angiopathy (CAA), which shares Aβ deposition with AD typical neurotic plaques that are localized within leptomeningeal and intracortical arteries, arterioles, and capillaries. CAA is commonly found in AD brains: up to approximately 50% of subjects with severe NP load [64]. CAA can affect perivascular drainage impairing glymphatic circulation, thus reducing a major route of Aβ clearance from the brain [59]. Intracranial atherosclerosis was found to be an additional, although not strictly neurodegenerative, strong risk factor for AD dementia [65].

## 4. Fluid Biomarkers: Ultrasensitive Measurement Techniques

Due to several advantages over invasive (e.g., CSF Aβ peptides), expensive and scarcely available (e.g., cerebral amyloid-PET) diagnostic tools, technologies aiming at quantifying NDDs biomarkers in blood are gathering momentum. 

However, the discovery of CNS-related biomarkers in blood presents challenging issues: (a) the concentration of a biomarker released in CNS is lower than in CSF, considering that it has to cross the blood–brain barrier and that the blood volume is consistently larger than the CSF one, (b) biomarkers could also be directly expressed peripherally, and the contribution of CNS might be difficult to quantify, (c) proteolytic degradation of the analytes by plasma proteases and confounding blood proteins may interfere with biomarker measurement [66]. 

The traditional enzyme-linked immunosorbent assay (ELISA) was extensively used in the last few decades. It showed a substantial intrinsic variability in the quantification of plasma/serum biomarkers and provided overlapping results in the discrimination between NDDs and cognitively healthy subjects [67,68]. The large sample volume required in the analysis combined with a sensitivity limited to the picomolar range could be addressed as the main weakness of this method. Therefore, ultrasensitive techniques often representing ELISA-based evolutions have been developed for blood biomarker discovery. 

The automated xMAP (multi-analyte profiling) Luminex technology, a flow cytometric method, allows the adaptation of several immunoassay formats to simultaneously detect multiple analytes on different sets of microspheres in a single well [69]. Through pre-made calibrators, it reduces measurement variability, partially overcoming some limitations of conventional ELISA methods (Table 1). 

Another emerging technique is the single-molecule array (Simoa), which is essentially a digital ELISA. This fully automated method is based on capturing antibody-coated paramagnetic beads loaded in arrays of femtomolar-sized reaction chambers with a volume 2 billion times smaller than conventional ELISA. Ultimately, by acquiring fluorescence images, an increase in signal will reflect the presence of single enzyme-associated immunocomplexes [70] (Table 1). This method is a candidate prescreening tool but may be potentially useful throughout the whole AD spectrum [71]. Large-scale longitudinal multicenter studies are anyway needed for the standardization and harmonization of preanalytical and analytical variables [72].

Combining the unique advantages of highly specific immunoreactions and electrochemiluminescence (ECL) biosensors, ECL immunoassays (ECLIA) have been implemented in several automated platforms. A wide dynamic detection range, low background noise, and simple optical set-ups are the strengths of this technique [73,74] (Table 1).

For protein analysis, immunoprecipitation has also been coupled with mass-spectrometry (IP-MS) providing a robust quantitative tool to identify antigens based on their intrinsic chemicophysical properties [75]. A significant advantage of this method is the possibility to analyze complex mixtures of Aβ peptides at very low concentrations in a single assay. However, multi-step structured analysis strategies are required in order to reduce the influence of non-specific binders and improve the signal quality (Table 1) [76]. A tailored approach for the identification of oligomers has been adopted in the Multimer Detection System (MDS), which is a modified sandwich ELISA originally designed to detect prion proteins. As opposed to the conventional method, this strategy relies on two epitope-overlapping antibodies for capturing and detecting an epitope, so that only multimers will bind to both antibodies, allowing their selective detection over monomers, which conversely will only bind to one of them (Table 1) [77,78]. 

Among the recently developed biosensors in AD research, the immuno-infrared sensor represents a promising label-free technique not aiming at discriminating particular Aβ species, but rather aiming at identifying the secondary structure distribution of all misfolded peptides. Thus, it is potentially exploitable in a preclinical setting (Table 1) [79,80,81].

A further virtuous application of the immunoassay principles is part of the immunomagnetic reduction (IMR) technique, in which magnetic antibody-coated nanoparticles dispersed in aqueous solution oscillate under external multiple alternating current (AC) magnetic fields. The association of target molecules determines a reduction in the AC magnetic susceptibility of capturing nanoparticles that will be as high as the concentration of the analytes (Table 1) [82]. Compared to ELISA-based techniques (e.g., Simoa), this method does not make use of beads to purify or concentrate antigens, and it is virtually able to quantify smaller proteins in higher number. Whether this could represent an advantage to detect Aβ peptides in plasma is still to be elucidated (Table 1).

Huge efforts have been made to develop and refine these technologies. Nevertheless, there is an urgent need to promote unbiased cross-platform evaluations for an effective method standardization.

In view of a targeted-oriented approach to AD, the adoption of guidelines to systemize preanalytical and postanalytical procedures across laboratories, aiming at finding consensus on a high-performance scalable platform for the discovery and approval of blood biomarkers, would be strongly recommended. 

## 5. Biomarkers Tracking Amyloid Pathology

Accumulating evidence from the clinical research consistently supports that CSF Aβ_1–42_ peptide shows an inverse correlation with plaque load in the brain [97,98,99,100,101] and provides important diagnostic information throughout the continuum of the AD spectrum. Therefore, this biomarker is currently integrated in the diagnostic criteria of AD; it is used for subject selection in clinical trials and approved in medical practice as well [13,14,19]. On the contrary, CSF Aβ_1–40_ peptide alone, albeit prevailing over the other Aβ species in both CNS and periphery, showed no relevant correlation with AD dementia [76,87,88,89]. Notably, the ratio of CSF Aβ_1–42_/Aβ_1–40_ has been found to predict cortical amyloid-PET positivity more accurately than CSF Aβ_1–42_ alone [90,91,92], improving the discrimination of AD vs. non-AD demented patients (Table 2) [93,94].

Some investigations suggest that also the CSF Aβ_1–42_/Aβ_1–38_ ratio turned out to improve cerebral amyloid deposition compared with CSF Aβ_1–42_ alone [94,95,96]. In addition, in pharmacological trials, short Aβ peptides detection in CSF may help monitor patients receiving drugs that modulate γ-secretase (Table 2) [97,98]. Among Aβ species, Aβ oligomers that are likely to play a key role in AD pathogenesis could be potentially used as preclinical biomarkers in CSF. Unfortunately, the detection of Aβ species and Aβ oligomers is challenging due to their polymorphous and unstable nature. Moreover, their concentration in biofluid is low, and they compete with other proteins and Aβ monomers. For the aforementioned reasons, most of the existing techniques are not satisfactory and reporting conflicting results so far [99]. As it also emerged from the Olsson and colleagues meta-analysis in 2016, Aβ1_1–42_ and Aβ_1–40_ peptides measured in blood with traditional ELISA methods did not discriminate AD from healthy controls [95]. 

Undoubtedly, the validation of relatively new technologies in the last few years—e.g., Simoa [100], immunoprecipitation-mass spectrometry (IP-MS) assays [74], stable labeling kinetics protocols [101], multimer detection system (MDS) [102], xMAP technology [103], immuno-infrared sensor [104], electrochemiluminescence immunoassays (ECLIA) [105,106,107,108,109,110,111,112]—led to a significant increased sensitivity in amyloid peptides detection in periphery when compared to the conventional ELISA technique, with drastically lower concentrations (up to the femtolitre) in blood than in CSF [65]. Particularly, in a 2017 study based on an IP-MS method, the plasma Aβ1-42/Aβ1-40 ratio was significantly lower in amyloid-PET positive compared with amyloid-PET negative participants. This ratio reported a good accuracy in distinguishing the two populations [101] (Table 3) [113,114,115,116,117,118,119,120,121,122,123,124,125,126,127,128,129,130,131]. 

In 2018, Nakamura and colleagues used the same technique to measure Aβ peptides in plasma of cognitive normal individuals, MCI and AD with dementia subjects, finding higher levels of plasma Aβ_1–40_/Aβ_1–42_ ratio in amyloid-PET positive compared with amyloid-PET negative individuals [75]. Regardless of the clinical diagnosis, the ratio of Aβ_1–40_ and Aβ_1–42_ peptides (Aβ_1–40_/Aβ_1–42_), and that of Aβ precursor protein fragment (APP_669–711_) and Aβ_1–42_ (APP_669–711_/Aβ_1–42_), had an excellent diagnostic accuracy in discriminating cerebral amyloid-PET positive and amyloid-PET negative subjects (Table 3). Similarly, in another study including a cohort of subjective memory complainers, a condition at risk for AD, the plasma Aβ_1–40_/Aβ_1–42_ ratio, turned out to be the best predictor of cerebral amyloidosis among a series of tested candidate biomarkers (e.g., β-site amyloid precursor protein cleaving enzyme 1 or BACE1, t-tau, NFL [72] (Table 3). Additional investigations performed using the Simoa technique reported a moderate accuracy of Aβ_1–42_/Aβ_1–40_ ratio in identifying the amyloid status. The Aβ_1–42_/Aβ_1–40_ ratio was lower in amyloid-PET positive compared with amyloid-PET negative participants [122,132] (Table 3). A further study in which Aβ peptides concentrations were assessed with a fully automated ECLIA technique confirmed the good discriminative role of Aβ_1–42_/Aβ_1–40_ ratio, in both validation and discovery cohorts [92] (Table 3). Similarly, Hanon and colleagues in a large investigation including 1040 MCI or AD participants reported that plasma Aβ_1–42_ and Aβ_1–40_ concentrations assessed by means of a kit based on a multiplex xMAP technique were lower in AD than in both MCI and non amnestic MCI (naMCI), suggesting a gradual decrease of these peripheral biomarkers with the course of the disease, in accordance to previous findings [109,133,134]. Conversely, in some studies where IMR was used, plasma Aβ_1–42_ concentrations are higher in AD patients than in healthy subjects [95,121]. Lue and colleagues reported a moderate diagnostic accuracy of this plasma biomarker in one cohort and excellent in the other [121] (Table 3). Moreover, in a 2018 study carried out by Nabers and colleagues using an immuno-infrared sensor, when compared with controls, not only were the concentrations of β-sheet-enriched Aβ peptides higher in severe AD dementia patients, as previously demonstrated [80], but they were also higher in prodromal AD patients, reaching a good diagnostic accuracy in identifying the amyloid status assessed by PET (Table 3) [80]. A recently developed ELISA method detecting Aβ multimers from monomers (MDS) showed a good accuracy of plasma Aβ oligomers in distinguishing AD patients from healthy controls [118] (Table 3). In parallel, in a subsequent study in which traditional ELISA was applied, plasma BACE-1 increase has emerged as another surrogate hallmark of AD progression [135]. In an effort to assess the plasma concentrations of Aβ_1–38_, Aβ_1–40_ and Aβ_1–42_ simultaneously, Shahpasand-Kroner and colleagues found that the Aβ_1–42_/Aβ_1–40_ and Aβ_1–42_/Aβ_1–38_ ratios are significantly lower in patients with AD dementia than in patients with dementia due to other causes and have a good accuracy in differentiating the two groups [120] (Table 3). 

In summary, quite concordant results regarding low plasmatic concentrations of Aβ_1–42_ and low Aβ_1–42_ /Aβ_1–40_ ratio in AD are reported in most of the studies in which an ultrasensitive technique has been applied. Overall, recent data suggest a low plasma Aβ_1–42_ and Aβ_1–42_/Aβ_1–40_ ratio as being a specific feature of AD patients with a weak to moderate and a moderate to high concordance with amyloid-PET outcomes, respectively [136] (Table 2). Further investigations comparing the different ultrasensitive techniques in the same populations will provide more accurate information regarding advantages and drawbacks.

## 6. Biomarkers for Tau Pathology

Together with CSF Aβ_1–42_, CSF t-tau and p-tau are considered as core biomarkers for AD diagnosis [11,137], and they are currently used for subject selection in clinical trials. Both CSF tau species are higher in patients compared to non-demented individuals. P-tau is more specific than t-tau for AD pathology and plays a main role in differential diagnosis being substantially normal in non-AD dementias [138]. Recent studies have shown that CSF tau can predict disease progression in both cognitively unimpaired and MCI subjects (Table 2) [139,140,141]. In 1999, Hulstaert and colleagues reported that the combined measurements of CSF Aβ_1–42_ and tau had a better outcome than the individual biomarker in differentiating AD patients from controls and other dementias [142]. In an effort to establish the utility of both CSF t-tau/Aβ_1–42_ and p-tau/Aβ_1–42_ ratios, several authors ended up confirming those preliminary findings [98,143,144]. Moreover, the ability of both ratios to predict disease onset and progression was proven in other studies, including normal individuals and MCI subjects, respectively [140,145,146].

Since 2013, it has been reported that plasma t-tau levels, measured through an assay based on digital array technology, were higher in AD participants compared with both MCI and healthy subjects, but they did not show significant modifications in subsequent longitudinal evaluations (Table 2) [116,147]. Shortly thereafter, a large meta-analysis demonstrated the increase of plasma t-tau levels to be strongly associated with AD (Table 2) [115]. 

In the last four years, highly sensitive immunoassay techniques significantly implemented tau levels detection in peripheral blood. Indeed, Mattsson and colleagues to assess plasma t-tau concentration in two separate cohorts applied the Simoa technique. In AD patients compared with both MCI and healthy controls, an increase of plasma tau was demonstrated but with overlapping results. More interestingly, longitudinal evaluations revealed that high baseline levels of this biomarker were predictive of cognitive decline, higher atrophy rates at MRI, and hypometabolism at 18F-FDG-PET as well [148]. A longitudinal study carried out using the same technique found increased plasma tau levels associated with a higher risk of MCI and cognitive decline in MCI subjects, irrespective of the total Aβ-burden in the brain [123] (Table 3). In 2019, Park and colleagues highlighted that both plasma t-tau measured by the Simoa technique as well and the plasma t-tau/Aβ_42_ ratio positively correlated with cerebral tau-PET uptake. Moreover, plasma tau-related biomarkers concentrations were significantly higher in Tau-PET+ subjects compared with Tau-PET- subjects and could differentiate the two groups with good accuracy (Table 3). It is noteworthy that the plasma t-tau and t-tau/Aβ_42_ ratio could predict the cerebral accumulation of this misfolded protein after a 2-year follow-up [149]. A clear association between plasma and CSF levels of p-tau was also found in Aβ+ patients, including the presymptomatic stage (Aβ+ cognitively unimpaired), but not in Aβ− individuals [90]. Overall, these data suggest to some extent that plasma t-tau concentration is high in AD patients, but the substantial overlap with normal controls hinders its diagnostic utility (Table 2). Interestingly, an additional study found out that plasma p-tau concentrations improve diagnostic accuracy significantly compared to plasma t-tau by reaching a good capability in the discrimination of amyloid-positive and amyloid-negative subjects (Table 3) [124]. Moreover, plasma p-tau levels assessed by IMR have shown a good accuracy in differentiating unimpaired and MCI subjects [125] (Table 3). 

The potential diagnostic role of plasma p-tau has been outlined in three recent investigations. In a first study performed with a novel ECLIA technique, plasma p-tau concentrations not only could discriminate AD and frontotemporal lobar degeneration (FTLD) participants with good accuracy, but they also identified amyloid-PET positive participants among elderly and MCI, and they predicted the rate of cognitive decline in AD and MCI over a 2-year follow-up (Table 3) [91]. The second study including three separate cohorts with 589 individuals (controls, MCI, AD, and non-AD NDDs) revealed that plasma p-tau levels likewise assessed by means of the MSD platform increase with disease progression (from preclinical to frank dementia phases encompassing prodromal/MCI stage) and can distinguish AD dementia from non-AD dementia with excellent accuracy (Table 3). Furthermore, plasma p-tau concentration was more elevated in Aβ+ cognitively unimpaired individuals than in Aβ− ones and in Aβ+ MCI who progressed to AD dementia compared to those who did not convert [90]. These results were confirmed in another study involving four independent cohorts (1131 total subjects) in different clinical contexts. Actually, plasma p-tau concentration measured by the Simoa technique discriminated AD dementia patients from both cognitively unimpaired subjects and other NDDs (including tauopathies such as Progressive Supranuclear Palsy and Corticobasal Syndrome) with optimal diagnostic accuracy (Table 3). Moreover, plasma p-tau predicted future cognitive decline over time. Interestingly, plasma p-tau concentration strongly correlates with cerebral amyloid-PET burden, even in amyloid-PET positive but tau-PET negative subjects, suggesting its crucial role in detecting the earliest disease phases. Thus, this biomarker might represent a screening tool implementable in different clinical settings and contexts of use [127].

## 7. Biomarkers for Neuroinflammation

The role of inflammation in AD pathogenesis was first suggested more than 20 years ago. Microglia, astrocytes, cytokines, and chemokines play a central role in disease pathogenesis since early phases [45,150]. Furthermore, amyloid and tau accumulation is linked to neuroinflammation [46,49,50,151], and Aβ accumulation evokes an exaggerated or heightened microglial response inducing and amplifying inflammatory reactions [152]. Therefore, biomarkers of neuroinflammation are gaining momentum in preclinical stages of AD and are useful to establish the eligibility of patients into new clinical trials [153,154,155,156]. 

A potential biomarker of neuroinflammation is the microglia/astrocyte-expressed protein YKL-40. YKL-40 is 45 a glycoprotein belonging to the family of 18 glycosyl hydrolases, and it is alternatively named human cartilage glycoprotein-39 (HC gp-39) or chitinase-3-like-1 protein (CHI3L1) [157]. CSF YKL-40 concentration is able to differentiate patients with typical AD dementia from cognitively normal controls with fair diagnostic accuracy [158,159]. Limited data regarding the ability of CSF YKL-40 to discriminate different NDDs are available so far. Actually, CSF YKL-40 differentiated AD from DLB, PD [160], FTLD [161], and non-AD MCI [162] with only a moderate diagnostic accuracy. Furthermore, CSF YKL-40 concentration is higher in AD versus Aβ-positive MCI subjects [163], and it significantly increases over time in the former (Table 2) [163]. CSF YKL-40 showed no ability in differentiating stable and progressing MCI [164,165], although it may predict progression to overt dementia in MCI [165] (Table 2). CSF YKL-40 levels negatively correlated with cortical thickness in specific AD-vulnerable areas, such as middle and inferior temporal areas in Aβ42-positive subjects [166] and grey matter volume in *APOE* ε4 carriers [167]. Interestingly, a positive association between CSF YKL-40 and t-tau has been reported in asymptomatic preclinical stages of AD and other NDDs [110,168], thus suggesting a link of YKL-40 with an underlying tau-driven neurodegenerative mechanisms [169]. 

YKL-40 has also been investigated in plasma, and elevated levels have been reported in patients with mild AD [170] and early AD [171] compared with controls. Unfortunately, plasma YKL-40 did not, so far, demonstrate utility as a diagnostic biomarker and for predicting cognitive decline (Table 2) [170,172]. To sum up, YKL-40 is an unspecific pathophysiological biomarker tracking immune/inflammatory response in NDDs, and it could be helpful as a monitoring biomarker for targeted anti-inflammatory therapies [169].

Another emerging biomarker of inflammation is “Triggering Receptor Expressed on Myeloid cells 2” (TREM2). TREM2 receptors play an important role in the pathogenesis of AD [173]. In the early stages of AD, TREM2 seems to be upregulated, probably in a protective intent [174]. However, due to the activation of inflammatory response, a detrimental role may prevail in later stages [175]. Some TREM2 genetic variants are related to AD possibly impairing microglia Aβ phagocytic ability and reducing, as a consequence, the cerebral Aß peptides clearance [167]. TREM2 has been proposed as AD biomarker in CSF, but with conflicting results so far. Some studies found higher CSF levels of TREM2 in AD [176,177,178,179] and MCI [176] compared to controls, and in subjects with MCI due to AD (or prodromal AD) compared with preclinical AD and AD dementia patients (Table 2) [179]. However, another study showed no difference between AD or MCI patients and cognitively normal controls [180]. A link between high CSF TREM2 value and neurodegeneration was proposed in MCI, considering that it positively correlated with gray matter volume and a negative correlation with mean diffusivity was detected [181]. Higher levels of TREM2 mRNA and TREM2 protein expressed in peripheral blood mononuclear cells were identified in AD patients compared to controls, with an inverse correlation with MMSE [182]. Moreover, TREM2 gene expression was found to be higher in MCI than AD patients [183]. Finally, a possible role of TREM2 as CSF and blood biomarker for AD has been suggested, but few data are currently available, and additional research is needed.

Another interesting candidate inflammatory biomarker is the monocyte chemoattractant protein-1 (MCP-1), which is a member of the C-C chemokine family and a potent chemotactic factor for monocytes [184]. Elevated CSF MCP-1 levels were found in AD patients compared to controls [185]. Noteworthy, also in blood, MCP-1 could be higher in MCI subjects than in controls [185]. 

Several other inflammatory biomarkers in CSF and blood have been investigated for their potential use as biomarkers for AD. A large metanalysis exploring inflammatory biomarkers in CSF demonstrated that AD patients could express higher levels of TGF-β compared with controls [350 Molievo]. TGF-ß1 is a neurotrophic, anti-inflammatory factor, and it enhances Aβ clearance by microglia activation. Since the early phases of disease, a reduced expression of TGF-ß has been described both in postmortem AD studies [186,187] and in animal models [188,189,190]. Two recently published meta-analyses investigating inflammatory biomarkers in blood reported an elevated tumor necrosis factor (TNF)-α, IL-12 [191], IL-1β, IL-2, IL-6, IL-18 [191,192], and reduced IL-1 receptor antagonist concentration in AD patients compared with controls. Mounting data about IL-6 as an AD biomarker are available. Blood IL-6 levels are associated with severity of cognitive decline in AD [193] and positively correlated with the cerebral ventricular volumes [194] and with matched CSF samples [195]. Blood IL-6 concentration was even higher in MCI individuals [135], suggesting that this biomarker is altered also in prodromal AD stages.

## 8. Biomarkers for Synaptic Dysfunction

Synaptic dysfunction is a core feature of AD, occurring early in the disease course. Synaptic density is strictly correlated with cognitive impairment and with Aβ and tau accumulation in AD, thus suggesting a central role in the underlying neurodegenerative process [196]. Based on these observations, several synaptic proteins have been investigated as potential diagnostic and prognostic biomarkers in AD. These include the quite well-characterized Neurogranin (Ng) and other emerging biomarkers such as Synaptosomal-Associated Protein 25 (SNAP-25), Synaptotagmin 1 and 2 (SYT-1 and SYT-2), Neuropentraxin 2 (NPTX-2), and Growth Associated Protein 43 (GAP-43) [197,198].

Ng is a post-synaptic protein largely expressed in the excitatory neurons of the hippocampus and cerebral cortex that acts as a calcium-sensitive modulator of post-synaptic signaling pathways and of long-term potentiation (LTP) [199]. Two recently published meta-analyses reported higher CSF Ng levels in AD compared to MCI and normal controls, thus supporting a role of CSF Ng as a useful diagnostic tool (Table 2) [200,201]. In particular, Ng reported good or even optimal diagnostic accuracy in differentiating AD patients with a full-blown clinical picture from cognitively normal subjects [202]. However, CSF Ng concentration discriminates between stable and converting MCI with poor diagnostic accuracy [200,201,203]. As regards its prognostic value, higher baseline CSF Ng levels are detected in controls and in MCI subjects who will convert to AD compared to non-converters, indicating a role of Ng in predicting progression to AD dementia in both cognitively normal [204] and MCI individuals (Table 2) [205,206]. CSF Ng could be also a reliable biomarker in the diagnostic workup of dementia being specifically more elevated in AD than non-AD dementias (FTD, DLB, but also VaD) [168,207,208,209,210]. Intriguingly, CSF Ng levels are high in AD patients with a typical amnesic phenotype, suggesting its role in the stratification and identification of AD subtypes, as a selective indicator of hippocampal degeneration [207]. In addition to Ng, other synaptic proteins have been explored as candidate biomarkers for AD. In particular, SNAP-25, a pre-synaptic protein involved in vesicle docking and neurotransmitter release, showed good accuracy in differentiating AD and MCI from normal controls (Table 2) [211,212,213]. Furthermore, high baseline CSF SNAP-25 levels predict future conversion to AD in MCI individuals [212]. Other pre-synaptic proteins such as SYT-1, SYT-2, NPTX-2, and GAP-43 are candidate as biomarkers to differentiate AD, MCI, and cognitive normal controls [197,213]. Importantly, an inverse correlation between CSF and neuron-derived plasma exosomes (NDE) levels of Ng, SYT-2, and GAP-43 has been observed [200,214]. Even if these results would deserve further supporting evidence, NDE may represent a window on the early synaptic dysfunction in AD and pave the way to a minimally invasive assessment (blood sample) of synaptic biomarkers in cognitively impaired and unimpaired subjects.

## 9. Biomarkers of Neuronal Injury

NFL is a subunit of neurofilaments that are neural cytoplasmic proteins designated to the structural stability of neurons; they are present in dendrites, soma, and also in axons. Axons physiologically release a low amount of NFL proteins that increase with aging [215]. The concentration of NFL significantly increases in both CSF and in blood as a result of axonal injury or neurodegeneration [216,217,218,219]. NFL in CSF is usually measured by sandwich ELISA technology. On the contrary, blood NFL concentration is 40-fold lower than in CSF, and it is below the sensitivity of ELISA or electrochemiluminescence assay technology [215]. Promising results came from recently developed ultrasensitive techniques capable of detecting even low concentrations of NFL in blood (Simoa) [220]. Despite being a sensitive biomarker of axonal injury, NFL is unspecific and did not discriminate between neurological diseases with a similar rate of axonal loss. However, growing data showed that CSF and, above all, blood NFL identifies neurodegeneration from early stages [215]. Indeed, NFL (CSF and blood) showed a good diagnostic accuracy in differentiating AD and FTD from healthy controls (Table 3) [128,129,131,208,221,222]. According to these results, a possible context-of-use of this biomarker is to rule out neurodegeneration in mimics such as psychiatric disturbances, or to early detect, within screening programs, the neurodegenerative process in a specific population at high risk (e.g., diabetes, elderly, genetic mutation carriers). Increased blood NFL concentration could also help clinicians to proceed or not with more invasive and expensive examinations in individuals with subjective memory complaints [215]. Moreover, CSF NFL but not t-tau, p-tau, and Ng might be a reliable risk biomarker being associated with a threefold higher risk to develop MCI over a median follow-up of 3.8 years in a population of cognitively healthy individuals [223]. CSF [224,225] and blood [128,226,227] NFL tightly correlated each other and with disease severity. In this regard, in a prospective case-control study including normal controls, MCI, and AD dementia patients, plasma NFL correlated with CSF NFL, poor cognition, cerebral atrophy, and brain hypometabolism [128].

Serum NFL concentration correlated with the estimated years to symptom onset and disease severity in autosomal dominant AD mutation carriers, suggesting its possible role as a risk biomarker in subjects with autosomal genetic mutations for AD (Table 3) [227]. Promising data concern the role of NFL in the differential diagnosis between FTD and AD. Actually, CSF NFL is higher in FTD patients compared to early onset AD, and the addition of NFL analysis improves the diagnostic accuracy of the traditional core biomarkers (p-tau181 and Aß42) up to a sensitivity of 86% and a specificity of 100% [228]. Similar findings were also reported in an autopsy-confirmed AD and FTD study (Table 3) [229]. Moreover, serum NFL could help in the differentiation of Primary Progressive Aphasia (PPA) variants. Indeed, serum NFL is increased in PPA compared to controls and discriminates between nfvPPA/svPPA (with a more likely FTD pathology) and lvPPA (where an AD pathology is expected in more than 50% of cases) with 81% and 67% of sensitivity and specificity, respectively [230]. 

Visinin-like protein 1 (VILIP-1) is emerging as a surrogate of signaling disruption and neuronal injury. VILIP-1 is a neuronal calcium sensor protein involved in signaling pathways related to synaptic plasticity [231]. A high intracellular concentration of Ca2+ induces the reversible translocation of VILIP-1 to the membrane components of the cell modulating signaling cascade in the neurons via the activation of specific membrane-bound targets [232]. The dysregulation of Ca2+ homeostasis is involved in AD neurodegeneration, bringing to a reduced intracellular expression of VILIP-1 and a quite selective damage of VILIP-1-containing neurons (cortical pyramidal cells, interneurons, septal, subthalamic, and hippocampal neurons) [232]. Therefore, this biomarker significantly increases in CSF [231]. Since VILIP-1 contributes to an altered Ca2+ homeostasis leading to neuronal loss [232], it is mainly considered a biomarker of neuronal injury. CSF VILIP-1 is higher in AD than in controls, but its diagnostic accuracy remains limited, especially in the prodromal stage (Table 2) [115,233,234]. Although VILIP-1 tightly correlated with p-tau and t-tau in CSF, conflicting results concern its relationship with Aß peptides [235,236]. CSF VILIP-1 and the VILIP-1/Aβ-42 ratio negatively correlate with MMSE and with the cerebral amyloid load, and they may predict a cognitive decline over time [233,234,236,237,238,239,240].

## 10. Toward Alternative Pathophysiological Pathways and Novel Matrices

The research on novel putative biomarkers in AD recently focused on two main directions: the exploration of new still under-characterized pathophysiological pathways, including mixed neuropathology models, and the identification of alternative easily accessible matrices.

TAR DNA-binding protein 43 (TDP43) is a DNA and RNA binding protein involved in transcription and splicing. TDP-43 contributes to neuroinflammation and may play a role in mitochondrial and neural dysfunction. In ALS and FTLD, its hyperphosphorylated and/or ubiquitinated cytoplasmic inclusions are detected [241,242] but also 20–50% of AD patients may show concomitant TDP-43 pathology [243,244,245]. Interestingly, TDP-43 pathology can be triggered by Aβ peptides [244]. In AD, increased plasma TDP-43 levels have been found compared to normal controls [246]; furthermore, plasma levels were increased also in the preclinical stage of subjects who subsequently progressed to AD dementia [247]. However, the evidence of a diagnostic and prognostic role of TDP-43 in AD is currently quite limited as well as its role in differentiating AD from other dementia mainly involving the hippocampus and memory (e.g., LATE) [22].

Lewy-related pathology (LRP), primarily consisting of α-synuclein (α-syn) aggregates, has been detected in more than half of autopsied AD brains, and higher levels of α-syn in the CSF of patients with MCI and AD have been associated with AD pathology and cognitive decline [248,249]. Moreover, CSF total α-syn (t-α-syn) and oligomeric α-synuclein (o-α-syn) levels were higher in AD [250] compared to PD, PD dementia and DLB individuals [251,252]. The use of standard ELISA methods to assess CSF α-syn levels does not ensure good diagnostic accuracy in discriminating AD from synucleinopathies [250]. Nevertheless, RT-QuIC [253] and protein misfolding cyclic amplification (PMCA) [254] are promising tools to identify AD individuals with α-syn co-pathology. Furthermore, growing interest toward the evaluation of α-syn heterocomplexes with Aβ_1–42_ (α-syn/Aβ) or tau (α-syn/tau) measured in red blood cells (RBCs) as peripheral pathophysiological markers of NDDs has been displayed [254]. Despite both α-syn alone, α-syn/Aβ and α-syn/tau heteroaggregates being found lower in AD compared to cognitive normal controls when isolated from red blood cells (RBC), only RBC α-syn/Aβ and α-syn/tau heterodimers discriminated AD from controls with fair accuracy [254].

Exploring alternative easily accessible matrices as a source of putative biomarkers is another key point of the search for novel fluid biomarkers. In this frame, exosomes represent an innovative and promising non-invasive tool to track early neurodegenerative changes occurring within the central nervous system. Exosomes are vesicles containing potential biomarkers for NDDs released into the extracellular space (that can be isolated from several body fluids) [243,254]. Proteins reflecting key events of the neurodegenerative process have been isolated in exosomes extracted from CSF and blood by using proteomic analysis [244,245,246]; in particular, p-tau was isolated in CSF exosomes from patients with mild AD (Braak stage 3) [247], and increased levels of exosomes-associated tau and Aβ were found in AD patients compared to controls [248]. Finally, other easily accessible matrices such as the retina may represent an open window on early neurodegenerative events in AD [249]. Amyloid pathology was demonstrated in the retina, and high-resolution non-invasive retinal imaging [47,48,49,50,51] represents an in vivo approach for visualizing Aβ deposits [250,251,252]. Indeed, retinal Aβ accumulation positively correlated with cerebral amyloid plaques [8]. Furthermore, decreased flow velocities in the retinal central veins were found in both MCI and AD compared to controls, thus suggesting a strict correlation with the underlying early neurodegenerative changes [253]. However, this field of research remains in its pathfinding stages, and a consensus on retinal imaging modalities, methodologies, and measures is still missing [253].

## 11. Conclusions

Recent research efforts are expanding the array of biomarkers on detecting and stratifying NDDs. Since 2007, fluid biomarkers have been reported within the diagnostic criteria of AD. In particular, Aß42 peptide, p-tau, and t-tau proteins measured in CSF became essential for a “modern” AD definition. The conceptual shift from a phenotype to a biomarker-based (or a precision medicine) diagnostic approach allowed the inclusion of the atypical subtypes within the AD spectrum and the exclusion of AD-mimics. For instance, patients with early and predominant behavioral impairment but positive for core pathophysiological biomarkers are categorized as AD and not as FTD. By contrast, individuals showing cognitive impairment of the hippocampal type but negative for core biomarkers are not considered AD. Definitely, the identification of a specific pathophysiological process in vivo by one or more biomarkers prevails on clinical phenotype. Unfortunately, validated fluid biomarkers used for AD diagnosis are invasive, time-consuming, expensive, not easily repeatable and, most importantly, not applicable as screening tools in large asymptomatic populations. On the other hand, the preclinical or prodromal identification of AD is urgent for patient recruitment in future disease-modifying treatments. This is an “expert” opinion based on the current literature, reporting the diagnostic and prognostic value of fluid biomarkers in AD. Five candidate molecules—three in plasma measured using ultrasensitive techniques (Aβ peptides, p-tau, and NFL proteins) and two in CSF (Ng and YKL-40)—with different potential context-of-use (Table 2) may be proposed. These molecules may enrich the current array of fluid biomarkers—CSF Aβ42, t-tau, and p-tau—for a more precise management of AD, and, broadly speaking, NDDs. These biomarkers are useful to both classify patients in different diagnostic categories and to track the pathophysiological mechanisms underlying neurodegeneration. The blood biomarkers (Aβ peptides, p-tau, NFL) are probably not more accurate than the respective molecules measured in CSF. However, they may be easily repeated over time, proposed in screening programs, and monitor treatments in disease-modifying trials. On the other hand, CSF YKL-40 and Ng are proxies of additional pathophysiological mechanisms related to AD, namely neuroinflammation and synaptic disruption, that cannot be efficiently evaluated with peripheral blood biomarkers. Therefore, CSF YKL-40 and Ng may be used in a subsequent diagnostic step to better stratify patients with prodromal or definite AD.

Plasma p-tau is increased in AD patients compared to controls and MCI individuals, discriminating AD demented patients from both cognitively unimpaired subjects and other NDDs with optimal diagnostic accuracy [91]. In several recent studies from different research groups, its classificatory accuracy surprisingly overlaps with cerebral amyloid-PET [90,124]. Moreover, plasma p-tau predicts a future cognitive decline over time [91]. Therefore, plasma p-tau, being easily repeatable, could be proposed in screening, diagnostic, prognostic, and monitoring context-of-uses. Simoa is the ultrasensitive technique used with more successful results across the studies on plasma p-tau so far. Of course, additional studies are needed. In addition, plasma t-tau concentration correlated with future cognitive decline, increased atrophy rates measured by MRI, and cerebral hypometabolism in FDG-PET images, but the results are less convincing and charted overlapping values among AD with dementia, prodromal, and preclinical AD groups [148].

Plasma Aβ peptides may represent a further significant improvement to facilitate the in vivo detection of amyloid pathology, substituting traditional core CSF biomarkers in next years. The combination of CSF traditional biomarkers (e.g., Aβ_1–42_/Aβ_1–40_ ratio, t-tau/Aβ_1–42_, and p-tau/Aβ_1–42_ ratios) can improve the diagnostic accuracy as well as the prediction of cognitive decline in AD patients. Similarly, the combination of plasma and serum biomarkers into ratios may increase the diagnostic power, although further evidence is needed [109,133]. Mounting data revealed that a low plasma Aβ_1–42_ and Aβ_1–42_/Aβ_1–40_ ratio are quite specific of AD pathology, although the concordance with cerebral amyloid-PET examination is variable and should be carefully evaluated in future studies [75,92,120]. In brief, further investigations should clarify in larger prospective studies: (1) the more accurate method to detect Aβ peptides and related by-products in plasma, (2) the pathophysiological role of plasma Aβ peptides and Aβ oligomers, and (3) their diagnostic and prognostic value as biomarker of AD.

NFL is mainly a marker of axonal degeneration and considered an unspecific indicator of neurodegeneration. Importantly, CSF and plasma NFL strictly correlated in all studies, suggesting that plasma NFL would be a reliable peripheral biomarker consistently reflecting modifications within the CNS. Indeed, many studies on NDDs demonstrated that diagnostic and prognostic accuracies of plasma and CSF NFL overlap [254]. NFL is a good example as a versatile biomarker for multiple context-of-use. It is useful to differentiate NDDs from mimics such as psychiatric disturbances or to early detect neurodegenerative processes in particular populations at risk (e.g., diabetes, elderly, genetic mutation carriers). NFL values were associated with a threefold higher risk to develop MCI, demonstrating a potential prognostic value [128,226,227]. Finally, the negative predictive value of plasma NFL might be used as a first step in screening programs for neurodegeneration, involving individuals with subjective memory complaints and late-onset psychiatric disorders. Concerning AD, plasma NFL showed a promising role in differentiating AD from bvFTD patients. It is likely that bvFTD individuals with an underlying TDP-43 pathology (related to amyotrophic lateral sclerosis) reported significantly higher plasma NFL value than AD subjects. Plasma NFL could early discriminate AD from more aggressive neurodegenerative dementia such as CJD [244]. Simoa was the only ultrasensitive technique used to measure plasma NFL in AD studies.

An increasing number of studies are focused on the development of precise biomarkers tracking additional key pathophysiological pathways leading to neurodegeneration, such as synaptic disruption and neuroinflammation. The pre-synaptic protein Ng measured in CSF is the most promising indicator of a synaptic dysfunction and hippocampal damage [202]. It could help stratify patients suffering from NDDs involving the hippocampus, including AD but also hippocampal sclerosis, LATE, and PART. LATE and PART have been recently defined in postmortem examinations, but in vivo diagnostic biomarkers are needed. CSF Ng could be also used as predictive indicator of an anticholinesterasic treatment response in patients showing a prevalent hippocampal impairment (typical AD phenotype, etc.) [200,201,205,206]. CSF Ng demonstrated from good to optimal diagnostic accuracy in discriminating AD dementia patients from the control group and a reliable prognostic value for AD conversion in MCI individuals.

Growing data reported an abnormal neuroinflammatory response in AD. Currently, CSF YKL-40 is the most promising fluid biomarker of glia activation, and it has been extensively investigated in other NDDs as well. Neuroinflammation is a common pathway of several NDDs, and not surprisingly, YKL-40 is an unspecific biomarker. This biomarker could be helpful in monitoring tailored anti-inflammatory trials in AD. Studies exploring a possible correlation between CSF YKL-40 concentration and cerebral inflammatory tracer as the translocator protein (TSPO)-PET uptake could clarify the role of this fluid biomarker as an indicator of neuroinflammation. CSF YKL-40 showed a fair diagnostic accuracy to discriminate AD patients from the control group and other neurodegenerative dementias. YKL-40 also reported a certain predictive value for MCI-AD progression.

In summary, we found that reliable fluid biomarkers might track three out of four of the main pathophysiological pathways of AD (Figure 1). The concentration in different biofluids of Aβ peptides and p-tau proteins reflect the cerebral misfolded protein deposition in AD. Moreover, plasma NFL might help the early identification of a general neurodegenerative process independently from the specific pathology. The diffusion of ultrasensitive techniques in the last few years is radically revolutionizing the context-of-use of these biomarkers in AD. The possibility to measure biomarkers in blood opens a completely novel scenario for the detection of multiple neurodegenerative mechanisms with a low cost and minimally invasive examination. This should encourage the development of screening tools in selected populations and improve the monitoring of disease-modifying trials. Of note, validated surrogates of co-pathologies such as α-syn and TDP-43 protein accumulation are currently not available as well as of cerebrovascular impairment. Finally, YKL-40 and Ng measured in CSF are promising proxies of neuroinflammation and synaptic disruption, respectively.

The studies described have several shortcomings. Many investigations reported different inclusion criteria and sometimes, they were not biomarker-based. Moreover, there is frequently a lack of data about comorbidities, especially cerebrovascular burden, contemporary pharmacological treatments, or stratification for age, gender, and genetic profiles [53]. Hepatic and kidney dysfunctions may impact biomarker levels as well as modifications of blood cell counts and plasma protein composition [67]. Nonetheless, these variables were not systematically considered, thus constituting a possible methodological bias, since individuals with AD present relevant vascular comorbidities.

From the prospective of a precision medicine approach, increasing attention is paid to find biomarkers associated to pathogenic pathways leading to neurodegeneration. The contemporary use of multiple biomarkers can help dissect the pathological mechanisms dynamically acting in space and time providing an accurate stratification of AD population.

AD is more a spectrum of different pathological mechanisms that brings a loss of proteostasis, which is the accumulation of several misfolded and aggregated proteins in multiple combinations rather than a single entity. Advanced statistical analysis, including unsupervised clustering strategies, combining clinical, biomarkers, and genetic data to collect subjects from large diversified cohorts into consistent clusters might be an innovative representation of AD [6] and other NDDs [254] and significantly contribute to the discovery of causes and tailored treatments. Novel data-driven classifications based on quantitative measurements of biomarkers and clinical information (e.g., standardized clinical scores) could improve the identification of effective and personalized therapies [253,254]. In conclusion, we assume that the identification and inclusion of AD patients in disease-modifying trials will be soon changed, mainly based on the demonstration of specific pathophysiological mechanisms and minimally influenced by phenotypes.

## Figures and Tables

**Table 1:** Key points of ultrasensitive techniques for the detection of putative blood biomarkers for AD.

METHOD	PROS	CONS
*xMAP*	It is a flexible technology with a workflow ranging from semi- to fully automated options. It enables the concomitant evaluation of multiple analytes in a single sample representing a time-, cost-, and labor-saving method. It enables a shift from a hypothesis-based analysis of known targets to a data-driven approach [83,84].	The simultaneous measurement of multiple ligands may favor cross-reactivities (“matrix effect”). A rigorous adherence to the manufacturer’s protocols is required to minimize any artifacts when using these kits [85].
*Simoa*	It is a fully automated technology based on antibody-coated paramagnetic microbeads. It has a great sensitivity (×1000 greater compared to conventional immunoassays), being able to detect single proteins at subfemtomolar concentrations. It is capable of multiplexing with short turnaround times and a remarkable throughput (up to 66 samples/h). It represents the most established ultrasensitive technology for blood biomarkers of AD to date (kits to measure Aβ1-42, p-tau181, t-tau, and NFL are available). A higher sensitivity compared to both ELISA and ECLIA-based methods was shown for the detection of NFL in serum [86,87,88].	Wide longitudinal multicenter studies are warranted for the standardization of preanalytical and analytical protocols parameters [72,88].
*ECLIA (MSD, Elecsys)*	ECLIA-based methods are adopted in semi- to fully automated (MSD) and fully automated (Elecsys) platforms. MSD is a flexible multi-array technology enabling the detection of biomarkers in single and high throughput multiplex formats. It provides a high inter-laboratory reproducibility, low matrix effects, reliability and cost-effectiveness [73,74]. Aβ peptides measured with Elecsys showed among the best accuracies in predicting the Aβ status assessed by either amyloid-PET or CSF Aβ1-42/Aβ1-40 ratio when compared to other techniques [89]. MSD provides good to optimal accuracy regarding the discriminative role of plasma p-tau181 to detect AD [90,91].	The accuracy of the Aβ1-42 and Aβ1-40 Elecsys assays is still suboptimal and insufficient to enable the use of these techniques alone as clinical tests of Aβ positivity. Additional cross-evaluations are needed before these ECLIA-based methods can be recommended [89].
*IP-MS*	It is able to characterize and quantify peptides by introducing them into the mass spectrometer after isolation through antibody-driven immunoprecipitation. Using this technique, optimal discriminative accuracies in detecting AD were reached by the Aβ1-40/Aβ1-42 ratio measured in plasma [75].	Antibodies and solid matrices also isolate many non-specific “contaminants”. To reduce the interferences with the signals and increase specificity in the detection of the antigens, targeted precautions are recommended (e.g., two rounds of repeated processing during the immunoprecipitation) [75,76]. Compared to automated ELISA-based techniques, IP-MS is a labor-intensive, low-throughput and time-consuming method not easily implementable on a wide scale [92].
*MDS*	It is an ELISA-based sandwich assay aiming at measuring oligomerization tendency in blood. It uses capture antibodies and epitope-overlapping detection antibodies to identify oligomers or multimers [93].	Its sensitivity in detecting Aβ oligomers failed to reach the cut-off of >80% that is needed for the validation of a biomarker [94].
*Immuno-infrared sensor*	It is an antibody-based method to extract all the Aβ peptides from blood samples, allowing the identification of β-sheet enriched conformations [79]. Compared to established ELISA-based tests, it does not measure the absolute biomarker concentration but the relative frequency shift in the infrared, reducing the influence of concentration fluctuations caused by biological variances [80]. Unique features of this assay are the absence of labels (enzymes, fluorescent or radioactive molecules) with potentially confounding effects, being the analytes detected based on their intrinsic physical properties, a simple and low-cost procedure and the low sample volume needed. It is able to identify the initial Aβ misfolding, occurring several years before clinical manifestation of AD [80].	Further tests in different clinical set-ups are needed to investigate the potential effects of sample handling and to evaluate their potential as screening-assays [79,80,81].
*IMR*	It measures the change in magnetic susceptibility caused by the association of antigens with antibody-coated paramagnetic nanobeads [82]. In contrast to ultrasensitive digital ELISA methodologies, IMR is a single-antibody immunoassay. Less stereoscopical interferences and a better ability to detect Aβ1-42 molecules in different conformations (isolated, complex or oligomeric forms) are strengths of this technique [95].	In regard to Aβ peptides, it provides results that are not consistent with those of the ELISA- and MS-based methods. The unspecific detection of Aβ aggregates or Aβ binding proteins likely caused by the single-antibody nature of the technique may explain the increase of plasma Aβ1-42 levels in AD patients compared to healthy controls [96].

*Abbreviations:* Aβ: amyloid β; Aβ1-40: amyloid β-peptide 1-40; Aβ1-42: amyloid β-peptide 1-42; AD: Alzheimer’s disease; CSF: cerebrospinal fluid; ECLIA: electrochemiluminescence immunoassay; ELISA: enzyme-linked immunosorbent assay; IMR: immunomagnetic reduction; IP-MS: immunoprecipitation coupled with mass spectrometry; MDS: multimer detection system; MSD: meso scale discovery; NFL: neurofilament light chain; p-tau: phosphorylated-tau; Simoa: single molecule array; t-tau: total tau; xMAP: multi-analyte profiling.

**Table 2:** Overview on the possible context of use of fluid biomarkers in AD.

		Diagnostic Value			Prognostic Value	Monitoring Treatment
		Preclinical Phase	Prodromal Phase	Full-Blown Picture		
Amyloid pathology						
Aβ peptides	Blood			+		
Aβ peptides	CSF		+	+	+	+
Tau pathology						
p-tau	Blood	+	+	+	+	
Neuroinflammation						
YKL-40	CSF			+	+	
Synaptic dysfunction						
Ng	CSF		+	+	+	
Neuronal structure and signaling disruption						
NFL	CSF		+	+	+	
	Blood		+	+	+	

Legend: plus sign (+): potential use, supportive data available. Abbreviations: Aβ: amyloid beta; t-tau: total tau; p-tau: phosphorylated-tau; YKL-40; Ng: neurogranin; NFL: neurofilaments; CSF: cerebrospinal fluid.

**Table 3:** Diagnostic and prognostic role of blood Aβ peptides, p-tau, t-tau, and NFL proteins measured with ultrasensitive techniques in AD.

Reference	Population	Study Design	Technique	Diagnostic Value	Prognostic Value
*Aβ peptides*					
Ovod V. et al., 2017 [113]	*N* = 41 (CU, AD dementia)	Longitudinal	IP-MS and stable labeling kinetics protocols	Aβ_1–42_/Aβ_1–40_ in differentiating amyloid positive participants vs. negative: AuROC = 0.89 with amyloid-PET and CSF Aβ_1–42_ as reference standards	NA
Wang M. et al., 2017 [114]	*N* = 61 (CU, AD dementia)	Cross-sectional	MDS	Aβ oligomers in differentiating AD patients vs. CU subjects: AuROC = 0.84 with clinical diagnosis (AD) as reference standard	NA
Lue L. et al., 2017 [115]	*N* = 124 (CU, AD dementia); U.S. cohort: *N* = 32; Taiwan cohort: *N* = 92	Cross-sectional	IMR	Aβ_1–42_ in differentiating AD patients vs. CU subjects: AuROC = 0.69 (U.S. cohort); AuROC = 0.96 (Taiwan cohort) with clinical diagnosis (AD) as reference standard	NA
Nakamura A et al., 2018 [74]	*N* = 484 (CU, MCI, AD)	Cross-sectional (retrospective)	IP-MS	APP/Aβ_1–42_ and Aβ_1–40_/Aβ_1–42_ in differentiating amyloid positive participants vs. negative: AuROC ≈0.90 compared with amyloid-PET as reference standard	NA
Nabers A. et al., 2018 [80]	*N* = 385 (CU, prodromal AD, AD); Sweden cohort: *N* = 73; Germany cohort: *N* = 312	Cross-sectional and nested case control	Immuno-infrared sensor	β-sheet-enriched Aβ peptides in differentiating: - amyloid positive participants vs. negative: AuROC = 0.78 (Sweden cohort) compared with amyloid-PET as reference standard; - AD vs. CU subjects: AuROC = 0.80 (Germany cohort)	NA
Shahpasand-Kroner H. et al., 2018 [120]	*N* = 40 (AD dementia, dementia due to other reasons)	Cross-sectional	ECLIA	Aβ_1–42_/Aβ_1–40_ in differentiating AD dementia vs. dementia due to other reasons: AuROC = 0.87 with clinical diagnosis as reference standard	NA
Verberk I. et al., 2018 [71]	*N* = 248 (SMC)	Longitudinal	Simoa	Aβ_1–42_/Aβ_1–40_ in differentiating amyloid positive SMC vs. negative: AuROC = 0.77 with CSF Aβ_1–42_ and amyloid PET as reference standards	Low Aβ_1–40_/Aβ_1–42_ is associated to MCI or dementia conversion (HR = 2.0) also after correcting for age and sex (HR=1.67)
Palmqvist S. et al., 2019 [89]	*N* = 1079 (CU, MCI, AD) Sweden cohort: *N* = 842 Germany cohort: *N* = 237	Multicenter and longitudinal	ECLIA	Aβ_1–42_ + Aβ_1–40_ (used as separate predictors in a logistic regression) in differentiating amyloid negative participants vs. positive: AuROC = 0.80 (Sweden cohort) and AuROC = 0.86 (Germany cohort) compared with CSF Aβ_1–42/_Aβ_1–40_ ratio as reference standard	NA
Vergallo A. et al., 2019 [72]	*N* = 276 (SMC)	Longitudinal	Simoa	Aβ_1–40_/Aβ_1–42_ in differentiating amyloid positive SMC vs. negative: AuROC = 0.77 compared with amyloid-PET as reference standard	NA
Chatterjee P. et al., 2019 [122]	*N* = 95 (CU)	Cross-sectional	Simoa	Aβ_1–40_/Aβ_1–42_ along with age and APOE ε4 status in differentiating amyloid positive participants vs. negative: AuROC = 0.78 compared with amyloid-PET as reference standard	NA
*p-tau and t-tau proteins*					
Mielke MM. et al., 2017 [123]	*N* = 458 (CU, MCI)	Longitudinal	Simoa		Both the middle (HR = 2.43) and the highest (HR = 2.02) tertiles of plasma t-tau levels are associated with increased risk of MCI in CU participants
Mielke MM. et al., 2018 [124]	*N* = 269 (CU, MCI, AD)	Cross-sectional	Simoa	In the discrimination between amyloid negative participants vs. positive: - plasma p-tau181: AuROC = 0.80; - plasma t-tau: AuROC = 0.60 compared with amyloid-PET as reference standard	NA
Yang C. et al., 2018 [125]	*N* = 73 (CU, MCI, very mild AD)	Cross-sectional	IMR	Plasma p-tau181 discriminating: - CU vs. MCI due to AD: AuROC = 0.85; - MCI due to AD vs. mild AD: AuROC = 0.78 with clinical diagnosis as reference standard	NA
Park JC. et al., 2019 [126]	*N* = 76 (CU, MCI, AD)	Both cross-sectional and longitudinal designs	Simoa (tau protein)/xMAP(Aβ_1–42_)	In the discrimination between tau positive participants vs. negative: - plasma t-tau/Aβ_1–42_ ratio: AuROC = 0.89; - plasma t-tau: AuROC = 0.80 with tau-PET as reference standard	NA
Janelidze S. et al., 2020 [90]	*N* = 589 (CU, MCI, AD dementia, non-AD dementia) cohort 1: *N* = 182 cohort 2: *N* = 344 cohort 3 (neuropathology cohort): *N* = 63	Both cross-sectional and longitudinal designs	ECLIA	Plasma p-tau181 in differentiating: - tau positive vs. negative participants: AuROC = 0.87–0.91 depending on different brain regions with tau-PET as reference standard (cohort 1); - AD dementia vs. non-AD dementia: AuROC = 0.94 with clinical diagnosis as reference standard (cohort 1); - Aβ positive vs. negative participants: AuROC ~ 0.80 (cohort 1 and cohort 2) with Aβ PET as reference standard; - AD dementia vs. non-AD dementia group: AuROC = 0.85 with neuropathology autopsy as reference standard (cohort 3)	High plasma p-tau levels are associated with future development of AD dementia in CU (HR = 2.48) and MCI (HR = 3.07) participants (cohort 2)
Thijssen E. et al., 2020 [91]	*N* = 404 (CU, MCI, AD, CBS, PSP, FTLD, nfvPPA, svPPA) 3 independent cohorts	Both cross-sectional (retrospective) and longitudinal designs	ECLIA	Plasma p-tau181 in differentiating: - AD (56) vs. FTLD (190) participants: AuROC = 0.89 with clinical diagnosis as reference standard; - Aβ-PET positive CU (11) vs. negative (29): AuROC = 0.86 with amyloid-PET as reference standard; - AD (15) vs. FTLD-tau participants (52): AuROC = 0.86 with neuropathology autopsy as reference standard	NA
Karikari T. et al., 2020 [127]	kari	Longitudinal	Simoa	Plasma p-tau181 in differentiating AD participants vs: - amyloid β negative young adults: AuROC = 0.99; - CU older adults: AuROC = 0.90–0.98 across cohorts; - vascular dementia participants: AuROC = 0.92; - PSP or CBS participants: AuROC = 0.89; - PD or MSA participants: AuROC = 0.82 with clinical diagnosis as reference standard - tau-PET positive vs. tau-PET negative individuals AuROC = 0.83–0.93 across cohorts with tau-PET as reference standard	NA
*NFL protein*					
Mattsson N. et al., 2017 [128]	*N* = 570 (CU, MCI, AD)	Case-control	Simoa	Plasma NFL in differentiating CU vs. AD participants: - AuROC = 0.87 with clinical diagnosis as reference standard	NA
Lewczuk P. et al., 2018 [129]	*N* = 99 (CU, MCI, AD)	Cross-sectional	Simoa	Plasma NFL in differentiating CU vs. diseased participants: - AuROC = 0.85 with clinical diagnosis as reference standard	NA
Steinacker P. et al., 2018 [130]	*N* = 132 (CU, MCI, AD, bvFTD)	Longitudinal	Simoa	Serum NFL in differentiating bvFTD vs: - AD: AuROC = 0.67 - MCI: AuROC = 0.90 - CU: AuROC = 0.85 with clinical diagnosis as reference standard Serum NFL in differentiating: - bvFTD vs. AD groups selected on the basis of CSF Aβ_1–42_ levels: AuROC = 0.79 - bvFTD vs. AD groups selected on the basis of both CSF Aβ_1–42_ and tau/p-tau levels: AuROC = 0.77	NA
Preische O. et al., 2019 [131]	*N* = 405 (controls - AD mutation non-carriers-, AD mutation carriers subdivided into presymptomatic mutation carriers, converters and symptomatic mutation carriers)	Longitudinal	Simoa	Rate of change of serum NFL in differentiating: - non-mutation carriers vs. presymptomatic mutation carriers: AuROC = 0.70 - non-mutation carriers vs. symptomatic non-mutation carriers: AuROC = 0.89 Baseline serum NFL levels in differentiating: - non-mutation carriers vs. presymptomatic mutation carriers: AuROC = 0.49 - non-mutation carriers vs. symptomatic non-mutation carriers: AuROC = 0.85	NA

*Abbreviations*: AD: Alzheimer’s disease; AuROC: area under the receiver operating curve; Aβ: amyloid β; Aβ_1–40_: amyloid β-peptide 1–40; Aβ_1–42_: amyloid β-peptide 1–42; bvFTD: behavioral variant frontotemporal dementia; CBS: corticobasal syndrome; CSF: cerebrospinal fluid; CU: cognitively unimpaired; ECLIA: electrochemiluminescence immunoassay; FTD: frontotemporal dementia; FTLD: frontotemporal lobar degeneration; HR: hazard ratio; IMR: immunomagnetic reduction; IP: immunoprecipitation; IP MS: immunoprecipitation coupled to mass spectrometry; MCI: mild cognitive impairment; MDS: multimer detection system; MS: mass spectrometry; MSA: multiple system atrophy; NA: not assessed; NFL: neurofilament light chain; nfvPPA: non-fluent variant primary progressive aphasia; PD: Parkinson’s disease; PPA: primary progressive aphasia; PSP: progressive supranuclear palsy; p-tau181: phospho-tau181; Simoa: single molecule array; SMC: subjective memory complainers; svPPA: semantic variant primary progressive aphasia; t-tau: total-tau; xMAP: multi-analyte profiling.

## References

[1] (2020). 2020 Alzheimer’s disease facts and figures. Alzheimer’s Dement..

[2] Schachter A.S., Davis K.L. (2000). Alzheimer’s disease. Curr. Treat. Options Neurol..

[3] Masters C.L., Simms G., Weinman N.A., Multhaup G., McDonald B.L., Beyreuther K. (1985). Amyloid plaque core protein in Alzheimer disease and Down syndrome. Proc. Natl. Acad. Sci. USA.

[4] McKhann G., Drachman D., Folstein M., Katzman R., Price D., Stadlan E.M. (1984). Clinical diagnosis of Alzheimer’s disease: Report of the NINCDS-ADRDA Work Group* under the auspices of Department of Health and Human Services Task Force on Alzheimer’s Disease. Neurology.

[5] Kovacs G.G. (2016). Molecular Pathological Classification of Neurodegenerative Diseases: Turning towards Precision Medicine. Int. J. Mol. Sci..

[6] Baldacci F., Lista S., Garaci F., Bonuccelli U., Toschi N., Hampel H. (2016). Biomarker-guided classification scheme of neurodegenerative diseases. J. Sport Health Sci..

[7] Jellinger K.A. (2010). Basic mechanisms of neurodegeneration: A critical update. J. Cell. Mol. Med..

[8] Baldacci F., Mazzucchi S., Della Vecchia A., Giampietri L., Giannini N., Koronyo-Hamaoui M., Ceravolo R., Siciliano G., Bonuccelli U., Elahi F.M. (2020). The path to biomarker-based diagnostic criteria for the spectrum of neurodegenerative diseases. Expert Rev. Mol. Diagn..

[9] Beach T.G., Monsell S.E., Phillips L.E., Kukull W. (2012). Accuracy of the Clinical Diagnosis of Alzheimer Disease at National Institute on Aging Alzheimer Disease Centers, 2005–2010. J. Neuropathol. Exp. Neurol..

[10] Dubois B., Feldman H.H., Jacova C., DeKosky S.T., Barberger-Gateau P., Cummings J.L., Delacourte A., Galasko D., Gauthier S., Jicha G. (2007). Research criteria for the diagnosis of Alzheimer’s disease: Revising the NINCDS–ADRDA criteria. Lancet Neurol..

[11] Jack C.R., Albert M.S., Knopman D.S., McKhann G.M., Sperling R.A., Carrillo M.C., Thies W., Phelps C.H. (2011). Introduction to the recommendations from the National Institute on Aging-Alzheimer’s Association workgroups on diagnostic guidelines for Alzheimer’s disease. Alzheimer’s Dement..

[12] Dubois B., Feldman H.H., Jacova C., Cummings J.L., DeKosky S.T., Barberger-Gateau P., Delacourte A., Frisoni G., Fox N.C., Galasko D. (2010). Revising the definition of Alzheimer’s disease: A new lexicon. Lancet Neurol..

[13] Dubois B., Feldman H.H., Jacova C., Hampel H., Molinuevo J.L., Blennow K., DeKosky S.T., Gauthier S., Selkoe D., Bateman R. (2014). Advancing research diagnostic criteria for Alzheimer’s disease: The IWG-2 criteria. Lancet Neurol..

[14] McKhann G.M., Knopman D.S., Chertkow H., Hyman B.T., Jack C.R., Kawas C.H., Klunk W.E., Koroshetz W.J., Manly J.J., Mayeux R. (2011). The diagnosis of dementia due to Alzheimer’s disease: Recommendations from the National Institute on Aging-Alzheimer’s Association workgroups on diagnostic guidelines for Alzheimer’s disease. Alzheimer’s Dement..

[15] Albert M.S., DeKosky S.T., Dickson D., Dubois B., Feldman H.H., Fox N.C., Gamst A., Holtzman D.M., Jagust W.J., Petersen R.C. (2011). The diagnosis of mild cognitive impairment due to Alzheimer’s disease: Recommendations from the National Institute on Aging-Alzheimer’s Association workgroups on diagnostic guidelines for Alzheimer’s disease. Alzheimer’s Dement..

[16] Sperling R.A., Aisen P.S., Beckett L.A., Bennett D.A., Craft S., Fagan A.M., Iwatsubo T., Jack C.R., Kaye J., Montine T.J. (2011). Toward defining the preclinical stages of Alzheimer’s disease: Recommendations from the National Institute on Aging-Alzheimer’s Association workgroups on diagnostic guidelines for Alzheimer’s disease. Alzheimer’s Dement..

[17] Dubois B., Hampel H., Feldman H.H., Scheltens P., Aisen P., Andrieu S., Bakardjian H., Benali H., Bertram L., Blennow K. (2016). Preclinical Alzheimer’s disease: Definition, natural history, and diagnostic criteria. Alzheimer’s Dement..

[18] Jack C.R., Bennett D.A., Blennow K., Carrillo M.C., Feldman H.H., Frisoni G.B., Hampel H., Jagust W.J., Johnson K.A., Knopman D.S. (2016). A/T/N: An unbiased descriptive classification scheme for Alzheimer disease biomarkers. Neurology.

[19] Jack C.R., Bennett D.A., Blennow K., Carrillo M.C., Dunn B., Haeberlein S.B., Holtzman D.M., Jagust W., Jessen F., Karlawish J. (2018). NIA-AA Research Framework: Toward a biological definition of Alzheimer’s disease. Alzheimer’s Dement..

[20] Crary J.F., Trojanowski J.Q., Schneider J.A., Abisambra J.F., Abner E.L., Alafuzoff I., Arnold S.E., Attems J., Beach T.G., Bigio E.H. (2014). Primary age-related tauopathy (PART): A common pathology associated with human aging. Acta Neuropathol..

[21] Nelson P.T., Dickson D.W., Trojanowski J.Q., Jack C.R., Boyle P., Arfanakis K., Rademakers R., Alafuzoff I., Attems J., Brayne C. (2019). Limbic-predominant age-related TDP-43 encephalopathy (LATE): Consensus working group report. Brain.

[22] Toledo J.B., Arnold S.E., Raible K., Brettschneider J., Xie S.X., Grossman M., Monsell S.E., Kukull W.A., Trojanowski J.Q. (2013). Contribution of cerebrovascular disease in autopsy confirmed neurodegenerative disease cases in the National Alzheimer’s Coordinating Centre. Brain.

[23] Hamilton R.L. (2006). Lewy Bodies in Alzheimer’s Disease: A Neuropathological Review of 145 Cases Using α-Synuclein Immunohistochemistry. Brain Pathol..

[24] Schneider J.A., Arvanitakis Z., Bang W., Bennett D.A. (2007). Mixed brain pathologies account for most dementia cases in community-dwelling older persons. Neorology.

[25] Colom-Cadena M., Grau-Rivera O., Planellas L., Cerquera C., Morenas E., Helgueta S., Muñoz L., Kulisevsky J., Martí M.J., Tolosa E. (2017). Regional Overlap of Pathologies in Lewy Body Disorders. J. Neuropathol. Exp. Neurol..

[26] Irwin D.J., Xie S.X., Coughlin D., Nevler N., Akhtar R.S., McMillan C.T., Lee E.B., Wolk D.A., Weintraub D., Chen-Plotkin A. (2018). CSF tau and β-amyloid predict cerebral synucleinopathy in autopsied Lewy body disorders. Neorology.

[27] Parnetti L., Paciotti S., Farotti L., Bellomo G., Sepe F.N., Eusebi P. (2019). Parkinson’s and Lewy body dementia CSF biomarkers. Clin. Chim. Acta.

[28] Van Bulck M., Sierra-Magro A., Alarcon-Gil J., Perez-Castillo A., Morales-García J.A. (2019). Novel Approaches for the Treatment of Alzheimer’s and Parkinson’s Disease. Int. J. Mol. Sci..

[29] Masters C.L., Bateman R., Blennow K., Rowe C.C., Sperling R.A., Cummings J.L. (2015). Alzheimer’s disease. Nat. Rev. Dis. Prim..

[30] Scheltens P., Blennow K., Breteler M.M.B., de Strooper B., Frisoni G.B., Salloway S., Van der Flier W.M. (2016). Alzheimer’s disease. Lancet.

[31] Hardy J., Higgins G., Mayford M., Barzilai A., Keller F., Schacher S., Kandel E. (1992). Alzheimer’s disease: The amyloid cascade hypothesis. Sci..

[32] Selkoe D.J., Hardy J. (2016). The amyloid hypothesis of Alzheimer’s disease at 25 years. EMBO Mol. Med..

[33] Christopher D., Guo J.L., McBride J.D., Narasimhan S., Kim H., Changolkar L., Zhang B., Gathagan R.J., Yue C., Dengler C. (2018). Amyloid-β plaques enhance Alzheimer’s brain tau-seeded pathologies by facilitating neuritic plaque tau aggregation. Nat. Med..

[34] Bakota L., Brandt R. (2016). Tau Biology and Tau-Directed Therapies for Alzheimer’s Disease. Drugs.

[35] Lee V.M., Balin B.J., Otvos L., Trojanowski J.Q. (1991). A68: A major subunit of paired helical filaments and derivatized forms of normal Tau. Science.

[36] Irwin D.J. (2016). Tauopathies as clinicopathological entities. Park. Relat. Disord..

[37] Sanabria-Castro A., Alvarado-Echeverría I., Monge-Bonilla C. (2017). Molecular Pathogenesis of Alzheimer’s Disease: An Update. Ann. Neurosci..

[38] Zhang B., Carroll J., Trojanowski J.Q., Yao Y., Iba M., Potuzak J.S., Hogan A.-M.L., Xie S.X., Ballatore C., Smith A.B. (2012). The Microtubule-Stabilizing Agent, Epothilone D, Reduces Axonal Dysfunction, Neurotoxicity, Cognitive Deficits, and Alzheimer-Like Pathology in an Interventional Study with Aged Tau Transgenic Mice. J. Neurosci..

[39] Khan S.S., Bloom G.S. (2016). Tau: The Center of a Signaling Nexus in Alzheimer’s Disease. Front. Neurosci..

[40] Leschik J., Welzel A., Weissmann C., Eckert A., Brandt R. (2007). Inverse and distinct modulation of tau-dependent neurodegeneration by presenilin 1 and amyloid-beta in cultured cortical neurons: Evidence that tau phosphorylation is the limiting factor in amyloid-?-induced cell death. J. Neurochem..

[41] Fath T., Eidenmüller J., Brandt R. (2002). Tau-Mediated Cytotoxicity in a Pseudohyperphosphorylation Model of Alzheimer’s Disease. J. Neurosci..

[42] Rogers J., Webster S., Lue L.-F., Brachova L., Civin W.H., Emmerling M., Shivers B., Walker D., McGeer P. (1996). Inflammation and Alzheimer’s disease pathogenesis. Neurobiol. Aging.

[43] Hull M., Berger M., Bauer J., Strauss S., Volk B. (1996). Inflammatory mechanisms in Alzheimer’s disease. Eur. Arch. Psychiatry Clin. Neurosci..

[44] Eikelenboom P. (1997). Neuroinflammation and Alzheimer’s Disease. Neurochemistry.

[45] Nordengen K., Kirsebom B.-E., Henjum K., Selnes P., Gísladóttir B., Wettergreen M., Torsetnes S.B., Grøntvedt G.R., Waterloo K.K., Aarsland D. (2019). Glial activation and inflammation along the Alzheimer’s disease continuum. J. Neuroinflamm..

[46] Morgan A.R., Touchard S., Leckey C., O’Hagan C., Nevado-Holgado A.J., Barkhof F., Bertram L., Blin O., Bos I., Dobricic V. (2019). Inflammatory biomarkers in Alzheimer’s disease plasma. Alzheimer’s Dement..

[47] Brosseron F., Krauthausen M., Kummer M., Heneka M.T. (2014). Body Fluid Cytokine Levels in Mild Cognitive Impairment and Alzheimer’s Disease: A Comparative Overview. Mol. Neurobiol..

[48] Fan Z., Brooks D.J., Okello A., Edison P. (2017). An early and late peak in microglial activation in Alzheimer’s disease trajectory. Brain.

[49] Edison P., Brooks D.J. (2018). Role of Neuroinflammation in the Trajectory of Alzheimer’s Disease and in vivo Quantification Using PET. J. Alzheimer’s Dis..

[50] Parbo P., Ismail R., Hansen K.V., Amidi A., Marup F., Gottrup H., Braendgaard H., Eriksson B., Eskildsen S., Lung T. (2017). Brain inflammation accompanies amyloid in the majority of mild cognitive impairment cases due to Alzheimer’s disease. Brain.

[51] Sarlus H., Heneka M.T. (2017). Microglia in Alzheimer’s disease. J. Clin. Investig..

[52] Hampel H., Caraci F., Cuello A.C., Caruso G., Nisticò R., Corbo M., Baldacci F., Toschi N., Garaci F., Chiesa P.A. (2020). A Path Toward Precision Medicine for Neuroinflammatory Mechanisms in Alzheimer’s Disease. Front. Immunol..

[53] Snyder H.M., Corriveau R.A., Craft S., Faber J.E., Greenberg S.M., Knopman D., Lamb B.T., Montine T.J., Nedergaard M., Schaffer C.B. (2015). Vascular contributions to cognitive impairment and dementia including Alzheimer’s disease. Alzheimer’s Dement..

[54] Zlokovic B.V. (2011). Neurovascular pathways to neurodegeneration in Alzheimer’s disease and other disorders. Nat. Rev. Neurosci..

[55] Kelleher R.J., Soiza R.L. (2013). Evidence of endothelial dysfunction in the development of Alzheimer’s disease: Is Alzheimer’s a vascular disorder?. Am. J. Cardiovasc. Dis..

[56] Salminen A., Kauppinen A., Kaarniranta K. (2017). Hypoxia/ischemia activate processing of Amyloid Precursor Protein: Impact of vascular dysfunction in the pathogenesis of Alzheimer’s disease. J. Neurochem..

[57] Di Marco L.Y., Venneri A., Farkas E., Evans P.C., Marzo A., Frangi A.F. (2015). Vascular dysfunction in the pathogenesis of Alzheimer’s disease — A review of endothelium-mediated mechanisms and ensuing vicious circles. Neurobiol. Dis..

[58] Tarasoff-Conway J.M., Carare R.O., Osorio R.S., Glodzik L., Butler T., Fieremans E., Axel L., Rusinek H., Nicholson C., Zlokovic B.V. (2015). Clearance systems in the brain—implications for Alzheimer disease. Nat. Rev. Neurol..

[59] Iliff J.J., Wang M., Liao Y., Plogg B.A., Peng W., Gundersen G.A., Benveniste H., Vates G.E., Deane R., Goldman S.A. (2012). A Paravascular Pathway Facilitates CSF Flow Through the Brain Parenchyma and the Clearance of Interstitial Solutes, Including Amyloid. Sci. Transl. Med..

[60] Xie L., Kang H., Xu Q., Chen M.J., Liao Y., Thiyagarajan M., O’Donnell J., Christensen D.J., Nicholson C., Iliff J.J. (2013). Sleep Drives Metabolite Clearance from the Adult Brain. Science.

[61] Attems J., Jellinger K. (2014). The overlap between vascular disease and Alzheimer’s disease - lessons from pathology. BMC Med..

[62] Snowdon D.A., Greiner L.H., Mortimer J.A., Riley K.P., Greiner P.A., Markesbery W.R. (1997). Brain Infarction and the Clinical Expression of Alzheimer DiseaseThe Nun Study. JAMA.

[63] Brenowitz W.D., Nelson P.T., Besser L.M., Heller K.B., Kukull W.A. (2015). Cerebral amyloid angiopathy and its co-occurrence with Alzheimer’s disease and other cerebrovascular neuropathologic changes. Neurobiol. Aging.

[64] Dolan H., Crain B., Troncoso J., Resnick S.M., Zonderman A.B., O’Brien R.J. (2010). Atherosclerosis, dementia, and alzheimer’s disease in the BLSA cohort. Ann. Neurol..

[65] Andreasson U., Blennow K., Zetterberg H. (2016). Update on ultrasensitive technologies to facilitate research on blood biomarkers for central nervous system disorders. Alzheimer’s Dement. Diagn. Assess. Dis. Monit..

[66] Baldacci F., Lista S., Vergallo A., Palermo G., Giorgi F.S., Hampel H. (2019). A frontline defense against neurodegenerative diseases:the development of early disease detection methods. Expert Rev. Mol. Diagn..

[67] Hampel H., O’Bryant S.E., Molinuevo J.L., Zetterberg H., Masters C.L., Lista S., Kiddle S.J., Batrla R., Blennow K. (2018). Blood-based biomarkers for Alzheimer disease: Mapping the road to the clinic. Nat. Rev. Neurol..

[68] Kang J.-H., Vanderstichele H., Trojanowski J.Q., Shaw L.M. (2012). Simultaneous analysis of cerebrospinal fluid biomarkers using microsphere-based xMAP multiplex technology for early detection of Alzheimer’s disease. Methods.

[69] Kuhle J., Barro C., Andreasson U., Derfuss T., Lindberg R., Sandelius Å., Liman V., Norgren N., Blennow K., Zetterberg H. (2016). Comparison of three analytical platforms for quantification of the neurofilament light chain in blood samples: ELISA, electrochemiluminescence immunoassay and Simoa. Clin. Chem. Lab. Med..

[70] Verberk I.M.W., Slot R.E., Verfaillie S.C.J., Heijst H., Prins N.D., Van Berckel B.N.M., Scheltens P., Teunissen C.E., Van Der Flier W.M. (2018). Plasma Amyloid as Prescreener for the Earliest A lzheimer Pathological Changes. Ann. Neurol..

[71] Vergallo A., Mégret L., Lista S., Cavedo E., Zetterberg H., Blennow K., Vanmechelen E., De Vos A., Habert M.-O., Potier M.-C. (2019). Plasma amyloid β 40/42 ratio predicts cerebral amyloidosis in cognitively normal individuals at risk for Alzheimer’s disease. Alzheimer’s Dement..

[72] Gowan S.M., Hardcastle A., Hallsworth A.E., Valenti M.R., Hunter L.-J.K., Brandon A.K.D.H., Garrett M.D., Raynaud F.I., Workman P., Aherne W. (2007). Application of Meso Scale Technology for the Measurement of Phosphoproteins in Human Tumor Xenografts. ASSAY Drug Dev. Technol..

[73] Fichorova R.N., Richardson-Harman N., Alfano M., Belec L., Carbonneil C., Chen S., Cosentino L., Curtis K., Dezzutti C.S., Donoval B. (2008). Biological and Technical Variables Affecting Immunoassay Recovery of Cytokines from Human Serum and Simulated Vaginal Fluid: A Multicenter Study. Anal. Chem..

[74] Nakamura A., Kaneko N., Villemagne V.L., Kato T., Doecke J., Doré V., Fowler C., Li Q.-X., Martins R., Rowe C. (2018). High performance plasma amyloid-β biomarkers for Alzheimer’s disease. Nat. Cell Biol..

[75] Have S.T., Boulon S., Ahmad Y., Lamond A.I. (2011). Mass spectrometry-based immuno-precipitation proteomics—The user’s guide. Proteomics.

[76] Seubert P., Vigo-Pelfrey C., Esch F., Lee M., Dovey H.F., Davis D., Sinha S., Schiossmacher M., Whaley J., Swindlehurst C. (1992). Isolation and quantification of soluble Alzheimer’s β-peptide from biological fluids. Nat. Cell Biol..

[77] Kim S.H., Kang S., Suh J.W., Park Y.H., Kang M.J., Pyun J.-M., Choi S.H., Jeong J.H., Park K.W., Lee H.-W. (2019). Blood amyloid-β oligomerization associated with neurodegeneration of Alzheimer’s disease. Alzheimer’s Res. Ther..

[78] Nabers A., Ollesch J., Schartner J., Kötting C., Genius J., Hafermann H., Klafki H., Gerwert K., Wiltfang J. (2016). Amyloid-β-Secondary Structure Distribution in Cerebrospinal Fluid and Blood Measured by an Immuno-Infrared-Sensor: A Biomarker Candidate for Alzheimer’s Disease. Anal. Chem..

[79] Nabers A., Perna L., Lange J., Mons U., Schartner J., Güldenhaupt J., Saum K., Janelidze S., Holleczek B., Rujescu D. (2018). Amyloid blood biomarker detects Alzheimer’s disease. EMBO Mol. Med..

[80] Nabers A., Hafermann H., Wiltfang J., Gerwert K. (2019). Aβ and tau structure-based biomarkers for a blood- and CSF-based two-step recruitment strategy to identify patients with dementia due to Alzheimer’s disease. Alzheimer’s Dement. Diagn. Assess. Dis. Monit..

[81] Yang S.-Y., Chiu M.-J., Chen T.-F., Horng H.-E. (2017). Detection of Plasma Biomarkers Using Immunomagnetic Reduction: A Promising Method for the Early Diagnosis of Alzheimer’s Disease. Neurol. Ther..

[82] Reslova N., Michna V., Kasny M., Mikel P., Kralik P. (2017). xMAP Technology: Applications in Detection of Pathogens. Front. Microbiol..

[83] Graham H., Chandler D.J., Dunbar S. (2019). The genesis and evolution of bead-based multiplexing. Methods.

[84] ElShal M.F., McCoy J.P. (2006). Multiplex bead array assays: Performance evaluation and comparison of sensitivity to ELISA☆. Methods.

[85] Wilson D.H., Rissin D.M., Kan C.W., Fournier D.R., Piech T., Campbell T.G., Meyer R.E., Fishburn M.W., Cabrera C., Patel P.P. (2016). The Simoa HD-1 Analyzer. J. Lab. Autom..

[86] Kuhle J., Barro C., Disanto G., Mathias A., Soneson C., Bonnier G., Yaldizli Ö., Regeniter A., Derfuss T., Canales M. (2016). Serum neurofilament light chain in early relapsing remitting MS is increased and correlates with CSF levels and with MRI measures of disease severity. Mult. Scler. J..

[87] Li D., Mielke M.M. (2019). An Update on Blood-Based Markers of Alzheimer’s Disease Using the SiMoA Platform. Neurol. Ther..

[88] Palmqvist S., Janelidze S., Stomrud E., Zetterberg H., Karl J., Zink K., Bittner T., Mattsson N., Eichenlaub U., Blennow K. (2019). Performance of Fully Automated Plasma Assays as Screening Tests for Alzheimer Disease–Related β-Amyloid Status. JAMA Neurol..

[89] Janelidze S., Mattsson N., Palmqvist S., Smith R., Beach T.G., Serrano G.E., Chai X., Proctor N.K., Eichenlaub U., Zetterberg H. (2020). Plasma P-tau181 in Alzheimer’s disease: Relationship to other biomarkers, differential diagnosis, neuropathology and longitudinal progression to Alzheimer’s dementia. Nat. Med..

[90] Thijssen E.H., La Joie R., Wolf A., Strom A., Wang P., Iaccarino L., Bourakova V., Cobigo Y., Heuer H., Advancing Research and Treatment for Frontotemporal Lobar Degeneration (ARTFL) investigators (2020). Diagnostic value of plasma phosphorylated tau181 in Alzheimer’s disease and frontotemporal lobar degeneration. Nat. Med..

[91] Wang M.J., Yi S., Han J.-Y., Park S.Y., Jang J.-W., Chun I.K., Kim S.E., Lee B.S., Kim G.J., Yu J.S. (2017). Oligomeric forms of amyloid-β protein in plasma as a potential blood-based biomarker for Alzheimer’s disease. Alzheimer’s Res. Ther..

[92] Teunissen C.E., Chiu M.-J., Yang C.-C., Yang S.-Y., Scheltens P., Zetterberg H., Blennow K. (2018). Plasma Amyloid-β (Aβ42) Correlates with Cerebrospinal Fluid Aβ42 in Alzheimer’s Disease. J. Alzheimer’s Dis..

[93] Zetterberg H. (2019). Blood-based biomarkers for Alzheimer’s disease—An update. J. Neurosci. Methods.

[94] Grimmer T., Riemenschneider M., Förstl H., Henriksen G., Klunk W.E., Mathis C.A., Shiga T., Wester H.-J., Kurz A., Drzezga A. (2009). Beta Amyloid in Alzheimer’s Disease: Increased Deposition in Brain Is Reflected in Reduced Concentration in Cerebrospinal Fluid. Biol. Psychiatry.

[95] Fagan A.M., Shaw L.M., Xiong C., Vanderstichele H., Mintun M.A., Trojanowski J.Q., Coart E., Morris J.C., Holtzman D.M. (2011). Comparison of Analytical Platforms for Cerebrospinal Fluid Measures of β-Amyloid 1-42, Total tau, and P-tau181 for Identifying Alzheimer Disease Amyloid Plaque Pathology. Arch. Neurol..

[96] Jagust W.J., Landau S.M., Shaw L.M., Trojanowski J.Q., Koeppe R.A., Reiman E.M., Foster N.L., Petersen R.C., Weiner M.W., Price J.C. (2009). Relationships between biomarkers in aging and dementia. Neurology.

[97] Strozyk D., Blennow K., White L.R., Launer L.J. (2003). CSF Aβ 42 levels correlate with amyloid-neuropathology in a population-based autopsy study. Neorology.

[98] Tapiola T., Alafuzoff I., Herukka S.-K., Parkkinen L., Hartikainen P., Soininen H., Pirttilä T. (2009). Cerebrospinal Fluid β-Amyloid 42 and Tau Proteins as Biomarkers of Alzheimer-Type Pathologic Changes in the Brain. Arch. Neurol..

[99] Suzuki N., Iwatsubo T., Odaka A., Ishibashi Y., Kitada C., Ihara Y. (1994). High tissue content of soluble β1-40 is linked to cerebral amyloid angiopathy. Am. J. Pathol..

[100] Mehta P.D., Pirttilä T., Mehta S.P., Sersen E.A., Aisen P.S., Wisniewski H.M. (2000). Plasma and cerebrospinal fluid levels of amyloid β proteins 1-40 and 1-42 in Alzheimer disease. Arch. Neurol..

[101] Slaets S., Le Bastard N., Martin J.-J., Sleegers K., Van Broeckhoven C., De Deyn P.P., Engelborghs S. (2013). Cerebrospinal Fluid Aβ1-40 Improves Differential Dementia Diagnosis in Patients with Intermediate P-tau181P Levels. J. Alzheimer’s Dis..

[102] Leuzy A., Chiotis K., Hasselbalch B.J., Rinne J.O., De Mendonça A., Otto M., Lleó A., Castelo-Branco M., Santana I., Johansson J. (2016). Pittsburgh compound B imaging and cerebrospinal fluid amyloid-β in a multicentre European memory clinic study. Brain.

[103] Lewczuk P., Matzen A., Blennow K., Parnetti L., Molinuevo J.L., Eusebi P., Kornhuber J., Morris J.C., Fagan A.M. (2017). Cerebrospinal Fluid Aβ42/40 Corresponds Better than Aβ42 to Amyloid PET in Alzheimer’s Disease. J. Alzheimer’s Dis..

[104] Pannee J., Portelius E., Minthon L., Gobom J., Andreasson U., Zetterberg H., Hansson O., Blennow K. (2016). Reference measurement procedure for CSF amyloid beta (Aβ)_1–42_ and the CSF Aβ _1–42_ /Aβ _1–40_ ratio—A cross-validation study against amyloid PET. J. Neurochem..

[105] Dorey A., Perret-Liaudet A., Tholance Y., Fourier A., Quadrio I. (2015). Cerebrospinal Fluid Aβ40 Improves the Interpretation of Aβ42 Concentration for Diagnosing Alzheimer’s Disease. Front. Neurol..

[106] Janelidze S., Stomrud E., Palmqvist S., Zetterberg H., Van Westen D., Jeromin A., Song L., Hanlon D., Hehir C.A.T., Baker D. (2016). Plasma β-amyloid in Alzheimer’s disease and vascular disease. Sci. Rep..

[107] Olsson B., Lautner R., Andreasson U., Öhrfelt A., Portelius E., Bjerke M., Hölttä M., Rosén C., Olsson C., Strobel G. (2016). CSF and blood biomarkers for the diagnosis of Alzheimer’s disease: A systematic review and meta-analysis. Lancet Neurol..

[108] Mulugeta E., Londos E., Ballard C., Alves G., Zetterberg H., Blennow K., Skogseth R., Minthon L., Aarsland D. (2010). CSF amyloid 38 as a novel diagnostic marker for dementia with Lewy bodies. J. Neurol. Neurosurg. Psychiatry.

[109] Olsson F., Schmidt S., Althoff V., Munter L.M., Jin S., Rosqvist S., Lendahl U., Multhaup G., Lundkvist J. (2013). Characterization of Intermediate Steps in Amyloid Beta (Aβ) Production under Near-native Conditions. J. Biol. Chem..

[110] Soares H.D., Gasior M., Toyn J.H., Wang J.-S., Hong Q., Berisha F., Furlong M.T., Raybon J., Lentz K.A., Sweeney F. (2016). The γ-secretase modulator, BMS-932481, modulates Aβ peptides in the plasma and cerebrospinal fluid of healthy volunteerss. J. Pharmacol. Exp. Ther..

[111] Schuster J., Funke S.A. (2016). Methods for the Specific Detection and Quantitation of Amyloid-β Oligomers in Cerebrospinal Fluid. J. Alzheimer’s Dis..

[112] Zetterberg H., Wilson D., Andreasson U., Minthon L., Blennow K., Randall J., Hansson O. (2013). Plasma tau levels in Alzheimer’s disease. Alzheimer’s Res. Ther..

[113] Ovod V., Ramsey K.N., Mawuenyega K.G., Bollinger J.G., Hicks T., Schneider T., Sullivan M., Paumier K., Holtzman D.M., Morris J.C. (2017). Amyloid β concentrations and stable isotope labeling kinetics of human plasma specific to central nervous system amyloidosis. Alzheimer’s Dement..

[114] Shahpasand-Kroner H., Klafki H.-W., Bauer C., Schuchhardt J., Hüttenrauch M., Stazi M., Bouter C., Wirths O., Vogelgsang J., Wiltfang J. (2018). A two-step immunoassay for the simultaneous assessment of Aβ38, Aβ40 and Aβ42 in human blood plasma supports the Aβ42/Aβ40 ratio as a promising biomarker candidate of Alzheimer’s disease. Alzheimer’s Res. Ther..

[115] Lue L.-F., Sabbagh M.N., Chiu M.-J., Jing N., Snyder N.L., Schmitz C., Guerra A., Belden C.M., Chen T.-F., Yang C.-C. (2017). Plasma Levels of Aβ42 and Tau Identified Probable Alzheimer’s Dementia: Findings in Two Cohorts. Front. Aging Neurosci..

[116] Chatterjee P., Elmi M., Goozee K., Shah T., Sohrabi H.R., Dias C.B., Pedrini S., Shen K., Asih P.R., Dave P. (2019). Ultrasensitive Detection of Plasma Amyloid-β as a Biomarker for Cognitively Normal Elderly Individuals at Risk of Alzheimer’s Disease. J. Alzheimer’s Dis..

[117] Mielke M.M., Hagen C.E., Wennberg A.M.V., Airey D.C., Savica R., Knopman D.S., Machulda M.M., Roberts R.O., Jack C.R., Petersen R.C. (2017). Association of Plasma Total Tau Level With Cognitive Decline and Risk of Mild Cognitive Impairment or Dementia in the Mayo Clinic Study on Aging. JAMA Neurol..

[118] Mielke M.M., Hagen C.E., Xu J., Chai X., Vemuri P., Lowe V.J., Airey D.C., Knopman D.S., Roberts R.O., Machulda M.M. (2018). Plasma phospho-tau181 increases with Alzheimer’s disease clinical severity and is associated with tau- and amyloid-positron emission tomography. Alzheimer’s Dement..

[119] Yang C.-C., Chiu M.-J., Chen T.-F., Chang H.-L., Liu B.-H., Yang S.-Y. (2018). Assay of Plasma Phosphorylated Tau Protein (Threonine 181) and Total Tau Protein in Early-Stage Alzheimer’s Disease. J. Alzheimer’s Dis..

[120] Park J.C., Han S.-H., Yi D., Byun M.S., Lee J.H., Jang S., Ko K., Jeon S.Y., Lee Y.-S., Kim Y.K. (2019). Plasma tau/amyloid-β1–42 ratio predicts brain tau deposition and neurodegeneration in Alzheimer’s disease. Brain.

[121] Karikari T.K., Pascoal T., Ashton N.J., Janelidze S., Benedet A.L., Rodriguez J.L., Chamoun M., Savard M., Kang M.S., Therriault J. (2020). Blood phosphorylated tau 181 as a biomarker for Alzheimer’s disease: A diagnostic performance and prediction modelling study using data from four prospective cohorts. Lancet Neurol..

[122] Mattsson N., Andreasson U., Zetterberg H., Blennow K., Alzheimer’s Disease Neuroimaging Initiative (2017). Association of Plasma Neurofilament Light with Neurodegeneration in Patients With Alzheimer Disease. JAMA Neurol..

[123] Lewczuk P., Ermann N., Andreasson U., Schultheis C., Podhorna J., Spitzer P., Maler J.M., Kornhuber J., Blennow K., Zetterberg H. (2018). Plasma neurofilament light as a potential biomarker of neurodegeneration in Alzheimer’s disease. Alzheimer’s Res. Ther..

[124] Steinacker P., Anderl-Straub S., Diehl-Schmid J., Semler E., Uttner I., Von Arnim C.A., Barthel H., Danek A., Fassbender K., Fliessbach K. (2018). Serum neurofilament light chain in behavioral variant frontotemporal dementia. Neurology.

[125] Hanon O., Vidal J.-S., Lehmann S., Bombois S., Allinquant B., Tréluyer J.-M., Gelé P., Delmaire C., Blanc F., Mangin J.-F. (2018). Plasma amyloid levels within the Alzheimer’s process and correlations with central biomarkers. Alzheimer’s Dement..

[126] Shen X.-N., Niu L.-D., Wang J., Cao X.-P., Liu Q., Tan L., Zhang C., Yu J.-T. (2019). Inflammatory markers in Alzheimer’s disease and mild cognitive impairment: A meta-analysis and systematic review of 170 studies. J. Neurol. Neurosurg. Psychiatry.

[127] Ashton N.J., Hye A., Rajkumar A.P., Leuzy A., Snowden S., Suárez-Calvet M., Karikari T.K., Schöll M., La Joie R., Rabinovici G.D. (2020). An update on blood-based biomarkers for non-Alzheimer neurodegenerative disorders. Nat. Rev. Neurol..

[128] Blennow K., Hampel H., Weiner M.W., Zetterberg H. (2010). Cerebrospinal fluid and plasma biomarkers in Alzheimer disease. Nat. Rev. Neurol..

[129] Roe C.M., Fagan A.M., Grant E.A., Hassenstab J., Moulder K.L., Dreyfus D.M., Sutphen C.L., Benzinger T.L., Mintun M.A., Holtzman D.M. (2013). Amyloid imaging and CSF biomarkers in predicting cognitive impairment up to 7.5 years later. Neurology.

[130] Ferreira D., Rivero-Santana A., Perestelo-Perez L., Westman E., Wahlund L.O., Sarría A., Serrano-Aguilar P. (2014). Improving CSF Biomarkersâ€™ Performance for Predicting Progression from Mild Cognitive Impairment to Alzheimer’s Disease by Considering Different Confounding Factors: A Meta-Analysis. Front. Aging Neurosci..

[131] Petersen R.C., Aisen P., Boeve B.F., Geda Y.E., Ivnik R.J., Knopman D.S., Mielke M., Pankratz V.S., Roberts R., Rocca W.A. (2013). Mild cognitive impairment due to Alzheimer disease in the community. Ann. Neurol..

[132] Hulstaert F., Blennow K., Ivanoiu A., Schoonderwaldt H.C., Riemenschneider M., Deyn P.P.D., Bancher C., Cras P., Wiltfang J., Mehta P.D. (1999). Improved discrimination of AD patients using -amyloid(1-42) and tau levels in CSF. Neurology.

[133] Rivero-Santana A., Ferreira D., Perestelo-Pérez L., Westman E., Wahlund L.O., Sarría A., Serrano-Aguilar P. (2017). Cerebrospinal Fluid Biomarkers for the Differential Diagnosis between Alzheimer’s Disease and Frontotemporal Lobar Degeneration: Systematic Review, HSROC Analysis, and Confounding Factors. J. Alzheimer’s Dis..

[134] Seeburger J.L., Holder D.J., Combrinck M., Joachim C., Laterza O., Tanen M., Dallob A., Chappell D., Snyder K., Flynn M. (2015). Cerebrospinal Fluid Biomarkers Distinguish Postmortem-Confirmed Alzheimer’s Disease from Other Dementias and Healthy Controls in the OPTIMA Cohort. J. Alzheimer’s Dis..

[135] Fagan A.M., Roe C.M., Xiong C., Mintun M.A., Morris J.C., Holtzman D.M. (2007). Cerebrospinal Fluid tau/β-Amyloid42 Ratio as a Prediction of Cognitive Decline in Nondemented Older Adults. Arch. Neurol..

[136] Van Rossum I., Vos S., Handels R.L.H., Visser P.J. (2010). Biomarkers as Predictors for Conversion from Mild Cognitive Impairment to Alzheimer-Type Dementia: Implications for Trial Design. J. Alzheimer’s Dis..

[137] Mattsson N., Zetterberg H., Janelidze S., Insel P.S., Andreasson U., Stomrud E., Palmqvist S., Baker D., Hehir C.A.T., Jeromin A. (2016). Plasma tau in Alzheimer disease. Neurology.

[138] Businaro R., Corsi M., Asprino R., Di Lorenzo C., Laskin D., Corbo R., Ricci S., Pinto A. (2018). Modulation of Inflammation as a Way of Delaying Alzheimer’s Disease Progression: The Diet’s Role. Curr. Alzheimer Res..

[139] Li J.-W., Zong Y., Cao X.-P., Tan L., Tan L. (2018). Microglial priming in Alzheimer’s disease. Ann. Transl. Med..

[140] Guzman-Martinez L., Maccioni R.B., Andrade V., Navarrete L.P., Pastor M.G., Ramos-Escobar N. (2019). Neuroinflammation as a Common Feature of Neurodegenerative Disorders. Front. Pharmacol..

[141] Hampel H., Vergallo A., Giorgi F.S., Kim S.H., Depypere H., Graziani M., Saidi A., Nisticò R., Lista S. (2018). Precision medicine and drug development in Alzheimer’s disease: The importance of sexual dimorphism and patient stratification. Front. Neuroendocr..

[142] Hampel H., Goetzl E.J., Kapogiannis D., Lista S., Vergallo A. (2019). Biomarker-Drug and Liquid Biopsy Co-development for Disease Staging and Targeted Therapy: Cornerstones for Alzheimer’s Precision Medicine and Pharmacology. Front. Pharmacol..

[143] Dhiman K., Blennow K., Zetterberg H., Martins R.N., Gupta V.B. (2019). Cerebrospinal fluid biomarkers for understanding multiple aspects of Alzheimer’s disease pathogenesis. Cell. Mol. Life Sci..

[144] Baldacci F., Lista S., Palermo G., Giorgi F.S., Vergallo A., Hampel H. (2019). The neuroinflammatory biomarker YKL-40 for neurodegenerative diseases: Advances in development. Expert Rev. Proteom..

[145] Llorens F., Thüne K., Tahir W., Kanata E., Diaz-Lucena D., Xanthopoulos K., Kovatsi E., Pleschka C., Garcia-Esparcia P., Schmitz M. (2017). YKL-40 in the brain and cerebrospinal fluid of neurodegenerative dementias. Mol. Neurodegener..

[146] Wennström M., Surova Y., Hall S., Nilsson C., Minthon L., Hansson O., Nielsen H.M. (2015). The Inflammatory Marker YKL-40 Is Elevated in Cerebrospinal Fluid from Patients with Alzheimer’s but Not Parkinson’s Disease or Dementia with Lewy Bodies. PLoS ONE.

[147] Baldacci F., Toschi N., Lista S., Zetterberg H., Blennow K., Kilimann I., Teipel S.J., Cavedo E., Dos Santos A.M., Epelbaum S. (2017). Two-level diagnostic classification using cerebrospinal fluid YKL-40 in Alzheimer’s disease. Alzheimer’s Dement..

[148] Hellwig K., Kvartsberg H., Portelius E., Andreasson U., Oberstein T.J., Lewczuk P., Blennow K., Kornhuber J., Maler J.M., Zetterberg H. (2015). Neurogranin and YKL-40: Independent markers of synaptic degeneration and neuroinflammation in Alzheimer’s disease. Alzheimer’s Res. Ther..

[149] Sutphen C.L., McCue L., Herries E.M., Xiong C., Ladenson J.H., Holtzman D.M., Fagan A.M., Adni O.B.O. (2018). Longitudinal decreases in multiple cerebrospinal fluid biomarkers of neuronal injury in symptomatic late onset Alzheimer’s disease. Alzheimer’s Dement..

[150] Zhang H., Ng K.P., Therriault J., Kang M.S., Pascoal T.A., Rosa-Neto P., Gauthier S. (2018). Cerebrospinal fluid phosphorylated tau, visinin-like protein-1, and chitinase-3-like protein 1 in mild cognitive impairment and Alzheimer’s disease 11 Medical and Health Sciences 1109 Neurosciences. Transl. Neurodegener..

[151] Janelidze S., Mattsson N., Stomrud E., Lindberg O., Palmqvist S., Zetterberg H., Blennow K., Hansson O. (2018). CSF biomarkers of neuroinflammation and cerebrovascular dysfunction in early Alzheimer disease. Neurology.

[152] Alcolea D., Vilaplana E., Pegueroles J., Montal V., Sanchez-Juan P., González-Suárez A., Pozueta A., Rodríguez-Rodríguez E., Bartrés-Faz D., Vidal-Pineiro D. (2015). Relationship between cortical thickness and cerebrospinal fluid YKL-40 in predementia stages of Alzheimer’s disease. Neurobiol. Aging.

[153] Gispert J.D., Monté G.C., Suárez-Calvet M., Falcon C., Tucholka A., Rojas S., Rami L., Sánchez-Valle R., Lladó A., Kleinberger G. (2017). The APOE ε4 genotype modulates CSF YKL-40 levels and their structural brain correlates in the continuum of Alzheimer’s disease but not those of sTREM2. Alzheimer’s Dement. Diagn. Assess. Dis. Monit..

[154] Janelidze S., Hertze J., Zetterberg H., Waldö M.L., Santillo A., Blennow K., Hansson O. (2015). Cerebrospinal fluid neurogranin and YKL -40 as biomarkers of Alzheimer’s disease. Ann. Clin. Transl. Neurol..

[155] Baldacci F., Lista S., Cavedo E., Bonuccelli U., Hampel H. (2017). Diagnostic function of the neuroinflammatory biomarker YKL-40 in Alzheimer’s disease and other neurodegenerative diseases. Expert Rev. Proteom..

[156] Craig-Schapiro R., Perrin R.J., Roe C.M., Xiong C., Carter D., Cairns N.J., Mintun M.A., Peskind E.R., Li G., Galasko D.R. (2010). YKL-40: A Novel Prognostic Fluid Biomarker for Preclinical Alzheimer’s Disease. Biol. Psychiatry.

[157] Choi J., Lee H.-W., Suk K. (2011). Plasma level of chitinase 3-like 1 protein increases in patients with early Alzheimer’s disease. J. Neurol..

[158] Vergallo A., Lista S., Lemercier P., Chiesa P.A., Zetterberg H., Blennow K., Potier M.-C., Habert M.-O., Baldacci F., Cavedo E. (2020). Association of plasma YKL-40 with brain amyloid-β levels, memory performance, and sex in subjective memory complainers. Neurobiol. Aging.

[159] Li J.-T., Zhang Y. (2018). TREM2 regulates innate immunity in Alzheimer’s disease. J. Neuroinflamm..

[160] Hickman S.E., El Khoury J. (2014). TREM2 and the neuroimmunology of Alzheimer’s disease. Biochem. Pharmacol..

[161] Jay T.R., Von Saucken V.E., Landreth G.E. (2017). TREM2 in Neurodegenerative Diseases. Mol. Neurodegener..

[162] Brosseron F., Traschütz A., Widmann C.N., Kummer M.P., Tacik P., Santarelli F., Jessen F., Heneka M.T. (2018). Characterization and clinical use of inflammatory cerebrospinal fluid protein markers in Alzheimer’s disease. Alzheimer’s Res. Ther..

[163] Heslegrave A.J., Heywood W., Paterson R.W., Magdalinou N.K., Svensson J., Johansson P., Öhrfelt A., Blennow K., Hardy J., Schott J.M. (2016). Increased cerebrospinal fluid soluble TREM2 concentration in Alzheimer’s disease. Mol. Neurodegener..

[164] Piccio L., Deming Y., Del-Águila J.L., Ghezzi L., Holtzman D.M., Fagan A.M., Fenoglio C., Galimberti D., Borroni B., Cruchaga C. (2016). Cerebrospinal fluid soluble TREM2 is higher in Alzheimer disease and associated with mutation status. Acta Neuropathol..

[165] Suárez-Calvet M., Kleinberger G., Caballero M., Ángel A., Brendel M., Rominger A., Alcolea D., Fortea J., Lleó A., Blesa R. (2016). sTREM 2 cerebrospinal fluid levels are a potential biomarker for microglia activity in early-stage Alzheimer’s disease and associate with neuronal injury markers. EMBO Mol. Med..

[166] Henjum K., Almdahl I.S., Årskog V., Minthon L., Hansson O., Fladby T., Nilsson L.N.G. (2016). Cerebrospinal fluid soluble TREM2 in aging and Alzheimer’s disease. Alzheimer’s Res. Ther..

[167] Gispert J.D., Suárez-Calvet M., Monté G.C., Tucholka A., Falcon C., Rojas S., Rami L., Sánchez-Valle R., Lladó A., Kleinberger G. (2016). Cerebrospinal fluid sTREM2 levels are associated with gray matter volume increases and reduced diffusivity in early Alzheimer’s disease. Alzheimer’s Dement..

[168] Hu N., Tan M.-S., Yu J.-T., Sun L., Tan L., Wang Y.-L., Jiang T., Tan L. (2013). Increased Expression of TREM2 in Peripheral Blood of Alzheimer’s Disease Patients. J. Alzheimer’s Dis..

[169] Casati M., Ferri E., Gussago C., Mazzola P., Abbate C., Bellelli G., Mari D., Cesari M., Arosio B. (2018). Increased expression of TREM2 in peripheral cells from mild cognitive impairment patients who progress into Alzheimer’s disease. Eur. J. Neurol..

[170] Deshmane S.L., Kremlev S., Amini S., Sawaya B.E. (2009). Monocyte Chemoattractant Protein-1 (MCP-1): An Overview. J. Interf. Cytokine Res..

[171] Ueberham U., Ueberham E., Gruschka H., Arendt T. (2006). Altered subcellular location of phosphorylated Smads in Alzheimer’s disease. Eur. J. Neurosci..

[172] Tesseur I., Zou K., Esposito L., Bard F., Berber E., Van Can J., Lin A.H., Crews L., Tremblay P., Mathews P. (2006). Deficiency in neuronal TGF-β signaling promotes neurodegeneration and Alzheimer’s pathology. J. Clin. Investig..

[173] Torrisi S.A., Geraci F., Tropea M.R., Grasso M., Caruso G., Fidilio A., Musso N., Sanfilippo G., Tascedda F., Palmeri A. (2019). Fluoxetine and Vortioxetine Reverse Depressive-Like Phenotype and Memory Deficits Induced by Aβ1-42 Oligomers in Mice: A Key Role of Transforming Growth Factor-β1. Front. Pharmacol..

[174] Caraci F., Spampinato S., Sortino M.A., Bosco P., Battaglia G., Bruno V., Drago F., Nicoletti F., Copani A. (2011). Dysfunction of TGF-β1 signaling in Alzheimer’s disease: Perspectives for neuroprotection. Cell Tissue Res..

[175] Liu C., Liu Q., Song L., Gu Y., Jie J., Bai X., Yang Y. (2014). Dab2 attenuates brain injury in APP/PS1 mice via targeting transforming growth factor-beta/SMAD signaling. Neural Regen. Res..

[176] Swardfager W., Lanctôt K., Rothenburg L., Wong A., Cappell J., Herrmann N. (2010). A Meta-Analysis of Cytokines in Alzheimer’s Disease. Biol. Psychiatry.

[177] Junttila A., Kuvaja M., Hartikainen P., Siloaho M., Helisalmi S., Moilanen V., Kiviharju A., Jansson L., Tienari P.J., Remes A.M. (2016). Cerebrospinal Fluid TDP-43 in Frontotemporal Lobar Degeneration and Amyotrophic Lateral Sclerosis Patients with and without the C9ORF72 Hexanucleotide Expansion. Dement. Geriatr. Cogn. Dis. Extra.

[178] Lai K.S.P., Liu C.S., Rau A., Lanctôt K.L., Köhler C., Pakosh M., Carvalho A.F., Herrmann N. (2017). Peripheral inflammatory markers in Alzheimer’s disease: A systematic review and meta-analysis of 175 studies. J. Neurol. Neurosurg. Psychiatry.

[179] Leung R., Proitsi P., Simmons A., Lunnon K., Güntert A., Kronenberg-Versteeg D., Pritchard M., Tsolaki M., Mecocci P., Kloszewska I. (2013). Inflammatory Proteins in Plasma Are Associated with Severity of Alzheimer’s Disease. PLoS ONE.

[180] Sun Y.-X., Minthon L., Wallmark A., Warkentin S., Blennow K., Janciauskiene S. (2003). Inflammatory Markers in Matched Plasma and Cerebrospinal Fluid from Patients with Alzheimer’s Disease. Dement. Geriatr. Cogn. Disord..

[181] Scheff S.W., Price D.A., Schmitt F.A., Mufson E.J. (2006). Hippocampal synaptic loss in early Alzheimer’s disease and mild cognitive impairment. Neurobiol. Aging.

[182] Galasko D., Xiao M., Xu D., Smirnov D., Salmon D.P., Dewit N., Vanbrabant J., Jacobs D., Vanderstichele H., Vanmechelen E. (2019). Synaptic biomarkers in CSF aid in diagnosis, correlate with cognition and predict progression in MCI and Alzheimer’s disease. Alzheimer’s Dement. Transl. Res. Clin. Interv..

[183] Molinuevo J.L., Ayton S., Batrla R., Bednar M.M., Bittner T., Cummings J., Fagan A.M., Hampel H., Mielke M.M., Mikulskis A. (2018). Current state of Alzheimer’s fluid biomarkers. Acta Neuropathol..

[184] Zhong L., Cherry T., E Bies C., Florence M., Gerges N.Z. (2009). Neurogranin enhances synaptic strength through its interaction with calmodulin. EMBO J..

[185] Liu W., Lin H., He X., Chen L., Dai Y., Jia W., Xue X., Tao J., Chen L. (2020). Neurogranin as a cognitive biomarker in cerebrospinal fluid and blood exosomes for Alzheimer’s disease and mild cognitive impairment. Transl. Psychiatry.

[186] Mavroudis I.A., Petridis F., Chatzikonstantinou S., Kazis D. (2019). A meta-analysis on CSF neurogranin levels for the diagnosis of Alzheimer’s disease and mild cognitive impairment. Aging Clin. Exp. Res..

[187] Mazzucchi S., Palermo G., Campese N., Galgani A., Della Vecchia A., Vergallo A., Siciliano G., Ceravolo R., Hampel H., Baldacci F. (2020). The role of synaptic biomarkers in the spectrum of neurodegenerative diseases. Expert Rev. Proteom..

[188] Mattsson N., Insel P.S., Palmqvist S., Portelius E., Zetterberg H., Weiner M., Blennow K., Hansson O., Initiative T.A.D.N. (2016). Cerebrospinal fluid tau, neurogranin, and neurofilament light in Alzheimer’s disease. EMBO Mol. Med..

[189] Tarawneh R., D’Angelo G., Crimmins D., Herries E., Griest T., Fagan A.M., Zipfel G.J., Ladenson J.H., Morris J.C., Holtzman D.M. (2016). Diagnostic and Prognostic Utility of the Synaptic Marker Neurogranin in Alzheimer Disease. JAMA Neurol..

[190] Kester M.I., Teunissen C.E., Crimmins D.L., Herries E.M., Ladenson J.H., Scheltens P., Van Der Flier W.M., Morris J.C., Holtzman D.M., Fagan A.M. (2015). Neurogranin as a Cerebrospinal Fluid Biomarker for Synaptic Loss in Symptomatic Alzheimer Disease. JAMA Neurol..

[191] Kvartsberg H., Duits F.H., Ingelsson M., Andreasen N., Öhrfelt A., Andersson K., Brinkmalm G., Lannfelt L., Minthon L., Hansson O. (2014). Cerebrospinal fluid levels of the synaptic protein neurogranin correlates with cognitive decline in prodromal Alzheimer’s disease. Alzheimer’s Dement..

[192] Wellington H., Paterson R.W., Portelius E., Törnqvist U., Magdalinou N., Fox N.C., Blennow K., Schott J.M., Zetterberg H. (2016). Increased CSF neurogranin concentration is specific to Alzheimer disease. Neurology.

[193] Lista S., Toschi N., Baldacci F., Zetterberg H., Blennow K., Kilimann I., Teipel S.J., Cavedo E., Santos A.M.D., Epelbaum S. (2017). Cerebrospinal fluid neurogranin as a biomarker of neurodegenerative diseases: A cross-sectional study. J. Alzheimer’s Dis..

[194] Portelius E., Olsson B., Höglund K., Cullen N.C., Kvartsberg H., Andreasson U., Zetterberg H., Sandelius Å., Shaw L.M., Lee V.M.Y. (2018). Cerebrospinal fluid neurogranin concentration in neurodegeneration: Relation to clinical phenotypes and neuropathology. Acta Neuropathol..

[195] Brinkmalm A., Brinkmalm G., Honer W.G., Frölich L., Hausner L., Minthon L., Hansson O., Wallin A., Zetterberg H., Blennow K. (2014). SNAP-25 is a promising novel cerebrospinal fluid biomarker for synapse degeneration in Alzheimer’s disease. Mol. Neurodegener..

[196] Zhang H., Initiative T.A.D.N., Therriault J., Kang M.S., Ng K.P., Pascoal T.A., Rosa-Neto P., Gauthier S. (2018). Cerebrospinal fluid synaptosomal-associated protein 25 is a key player in synaptic degeneration in mild cognitive impairment and Alzheimer’s disease. Alzheimer’s Res. Ther..

[197] Öhrfelt A., Brinkmalm A., Dumurgier J., Zetterberg H., Bouaziz-Amar E., Hugon J., Paquet C., Blennow K. (2019). A Novel ELISA for the Measurement of Cerebrospinal Fluid SNAP-25 in Patients with Alzheimer’s Disease. Neuroscience.

[198] Goetzl E.J., Kapogiannis D., Schwartz J.B., Lobach I.V., Goetzl L., Abner E.L., Jicha G.A., Karydas A.M., Boxer A., Miller B.L. (2016). Decreased synaptic proteins in neuronal exosomes of frontotemporal dementia and Alzheimer’s disease. FASEB J..

[199] Gaetani L., Blennow K., Calabresi P., Di Filippo M., Parnetti L., Zetterberg H. (2019). Neurofilament light chain as a biomarker in neurological disorders. J. Neurol. Neurosurg. Psychiatry.

[200] Brureau A., Blanchard-Bregeon V., Pech C., Hamon S., Chaillou P., Guillemot J.-C., Barneoud P., Bertrand P., Pradier L., Rooney T. (2017). NF-L in cerebrospinal fluid and serum is a biomarker of neuronal damage in an inducible mouse model of neurodegeneration. Neurobiol. Dis..

[201] Bacioglu M., Maia L.F., Preische O., Schelle J., Apel A., Kaeser S.A., Schweighauser M., Eninger T., Lambert M., Pilotto A. (2016). Neurofilament Light Chain in Blood and CSF as Marker of Disease Progression in Mouse Models and in Neurodegenerative Diseases. Neuron.

[202] Barro C., Benkert P., Disanto G., Tsagkas C., Amann M., Naegelin Y., Leppert D., Gobbi C., Granziera C., Yaldizli Ö. (2018). Serum neurofilament as a predictor of disease worsening and brain and spinal cord atrophy in multiple sclerosis. Brain.

[203] Disanto G., Barro C., Benkert P., Naegelin Y., Schädelin S., Giardiello A., Zecca C., Blennow K., Zetterberg H., Leppert D. (2017). Serum Neurofilament light: A biomarker of neuronal damage in multiple sclerosis. Ann. Neurol..

[204] Rohrer J.D., Woollacott I.O., Dick K.M., Brotherhood E., Gordon E., Fellows A., Toombs J., Druyeh R., Cardoso M.J., Ourselin S. (2016). Serum neurofilament light chain protein is a measure of disease intensity in frontotemporal dementia. Neurology.

[205] Alcolea D., Vilaplana E., Suárez-Calvet M., Illán-Gala I., Blesa R., Clarimón J., Lladó A., Sánchez-Valle R., Molinuevo J.L., García-Ribas G. (2017). CSF sAPPβ, YKL-40, and neurofilament light in frontotemporal lobar degeneration. Neurology.

[206] Kern S., Syrjanen J.A., Blennow K., Zetterberg H., Skoog I., Waern M., Hagen C.E., Van Harten A.C., Knopman D.S., Jack C.R. (2018). Association of Cerebrospinal Fluid Neurofilament Light Protein With Risk of Mild Cognitive Impairment Among Individuals Without Cognitive Impairment. JAMA Neurol..

[207] Skillbäck T., Farahmand B., Bartlett J.W., Rosén C., Mattsson N., Nägga K., Kilander L., Religa D., Wimo A., Winblad B. (2014). CSF neurofilament light differs in neurodegenerative diseases and predicts severity and survival. Neurology.

[208] Zetterberg H., Skillbäck T., Mattsson N., Trojanowski J.Q., Portelius E., Shaw L.M., Weiner M.W., Blennow K., Alzheimer’s Disease Neuroimaging Initiative (2016). Association of Cerebrospinal Fluid Neurofilament Light Concentration With Alzheimer Disease Progression. JAMA Neurol..

[209] Sánchez-Valle R., Heslegrave A., Foiani M.S., Bosch B., Antonell A., Balasa M., Lladó A., Toombs J., Fox N.C. (2018). Serum neurofilament light levels correlate with severity measures and neurodegeneration markers in autosomal dominant Alzheimer’s disease. Alzheimer’s Res. Ther..

[210] De Jong D., Jansen R.W.M.M., Pijnenburg Y.A.L., Van Geel W.J., Borm G.F., Kremer H.P.H., Verbeek M.M. (2007). CSF neurofilament proteins in the differential diagnosis of dementia. J. Neurol. Neurosurg. Psychiatry.

[211] Olsson B., Portelius E., Cullen N.C., Sandelius Å., Zetterberg H., Andreasson U., Höglund K., Irwin D.J., Grossman M., Weintraub D. (2019). Association of Cerebrospinal Fluid Neurofilament Light Protein Levels With Cognition in Patients With Dementia, Motor Neuron Disease, and Movement Disorders. JAMA Neurol..

[212] Steinacker P., Semler E., Anderl-Straub S., Diehl-Schmid J., Schroeter M.L., Uttner I., Foerstl H., Landwehrmeyer B., Von Arnim C.A., Kassubek J. (2017). Neurofilament as a blood marker for diagnosis and monitoring of primary progressive aphasias. Neorology.

[213] Groblewska M., Muszyński P., Wojtulewska-Supron A., Kulczyńska-Przybik A., Mroczko B. (2015). The Role of Visinin-Like Protein-1 in the Pathophysiology of Alzheimer’s Disease. J. Alzheimer’s Dis..

[214] Bernstein H.-G., Baumann B., Danos P., Diekmann S., Bogerts B., Gundelfinger E.D., Braunewell K.-H. (1999). Regional and cellular distribution of neural visinin-like protein immunoreactivities (VILIP-1 and VILIP-3) in human brain. J. Neurocytol..

[215] Mroczko B., Groblewska M., Zboch M., Muszyński P., Zajkowska A., Borawska R., Szmitkowski M., Kornhuber J., Lewczuk P. (2014). Evaluation of Visinin-Like Protein 1 Concentrations in the Cerebrospinal Fluid of Patients with Mild Cognitive Impairment as a Dynamic Biomarker of Alzheimer’s Disease. J. Alzheimer’s Dis..

[216] Leko M.B., Borovečki F., Dejanović N., Hof P.R., Šimić G. (2016). Predictive Value of Cerebrospinal Fluid Visinin-Like Protein-1 Levels for Alzheimer’s Disease Early Detection and Differential Diagnosis in Patients with Mild Cognitive Impairment. J. Alzheimer’s Dis..

[217] Braunewell K.H. (2012). The visinin-like proteins VILIP-1 and VILIP-3 in Alzheimer’s disease—old wine in new bottles. Front. Mol. Neurosci..

[218] Lee J.-M., Blennow K., Andreasen N., Laterza O., Modur V., Olander J., Gao F., Ohlendorf M., Ladenson J.H. (2008). The Brain Injury Biomarker VLP-1 Is Increased in the Cerebrospinal Fluid of Alzheimer Disease Patients. Clin. Chem..

[219] Luo X., Hou L., Shi H., Zhong X., Zhang Y., Zheng D., Tan Y., Hu G., Mu N., Chan J. (2013). CSF levels of the neuronal injury biomarker visinin-like protein-1 in Alzheimer’s disease and dementia with Lewy bodies. J. Neurochem..

[220] Tarawneh R.M., D’Angelo G., Ba E.M., Xiong C., Carter D., Cairns N.J., Fagan A.M., Head D., Mintun M.A., Ladenson J.H. (2011). Visinin-like protein-1: Diagnostic and prognostic biomarker in Alzheimer disease. Ann. Neurol..

[221] Tarawneh R.M., Head D., Allison S., Buckles V., Fagan A.M., Ladenson J.H., Morris J.C., Holtzman D.M. (2015). Cerebrospinal Fluid Markers of Neurodegeneration and Rates of Brain Atrophy in Early Alzheimer Disease. JAMA Neurol..

[222] Tarawneh R., Lee J.-M., Ladenson J.H., Morris J.C., Holtzman D.M. (2012). CSF VILIP-1 predicts rates of cognitive decline in early Alzheimer disease. Neurology.

[223] Chen-Plotkin A.S., Lee V.M.-Y., Trojanowski J.Q. (2010). TAR DNA-binding protein 43 in neurodegenerative disease. Nat. Rev. Neurol..

[224] Neumann M., Sampathu D.M., Kwong L.K., Truax A.C., Micsenyi M.C., Chou T.T., Bruce J., Schuck T., Grossman M., Clark C.M. (2006). Ubiquitinated TDP-43 in Frontotemporal Lobar Degeneration and Amyotrophic Lateral Sclerosis. Science.

[225] Amador-Ortiz C., Lin W.-L., Ahmed Z., Ms D.P., Davies P., Duara R., Graff-Radford N.R., Hutton M.L., Dickson D.W. (2007). TDP-43 immunoreactivity in hippocampal sclerosis and Alzheimer’s disease. Ann. Neurol..

[226] Chang X.-L., Tan M.-S., Tan L., Yu J.-T. (2015). The Role of TDP-43 in Alzheimer’s Disease. Mol. Neurobiol..

[227] James B.D., Wilson R.S., Boyle P.A., Trojanowski J.Q., Bennett D.A., Schneider J.A. (2016). TDP-43 stage, mixed pathologies, and clinical Alzheimer’s-type dementia. Brain.

[228] Foulds P., McAuley E., Gibbons L., Davidson Y., Pickering-Brown S., Neary D., Snowden J.S., Allsop D., Mann D.M.A. (2008). TDP-43 protein in plasma may index TDP-43 brain pathology in Alzheimer’s disease and frontotemporal lobar degeneration. Acta Neuropathol..

[229] Williams S.M., Schulz P., Rosenberry T.L., Caselli R.J., Sierks M.R. (2017). Blood-Based Oligomeric and Other Protein Variant Biomarkers to Facilitate Pre-Symptomatic Diagnosis and Staging of Alzheimer’s Disease. J. Alzheimer’s Dis..

[230] Vergallo A., Bun R.-S., Toschi N., Baldacci F., Zetterberg H., Blennow K., Cavedo E., Lamari F., Habert M.-O., Dubois B. (2018). Association of cerebrospinal fluid α-synuclein with total and phospho-tau181 protein concentrations and brain amyloid load in cognitively normal subjective memory complainers stratified by Alzheimer’s disease biomarkers. Alzheimer’s Dement..

[231] Twohig D., Rodriguez-Vieitez E., Sando S.B., Berge G., Lauridsen C., Møller I., Grøntvedt G.R., Bråthen G., Patra K., Dominantly Inherited Alzheimer Network (DIAN) (2018). The relevance of cerebrospinal fluid α-synuclein levels to sporadic and familial Alzheimer’s disease. Acta Neuropathol. Commun..

[232] Twohig D., Nielsen H.M. (2019). α-synuclein in the pathophysiology of Alzheimer’s disease. Mol. Neurodegener..

[233] Gao L., Tang H., Nie K., Wang L., Zhao J., Gan R., Huang J., Zhu R., Feng S., Duan Z. (2014). Cerebrospinal fluid alpha-synuclein as a biomarker for Parkinson’s disease diagnosis: A systematic review and meta-analysis. Int. J. Neurosci..

[234] Esako W., Murakami N., Izumi Y., Kaji R. (2014). Reduced alpha-synuclein in cerebrospinal fluid in synucleinopathies: Evidence from a meta-analysis. Mov. Disord..

[235] Fairfoul G., McGuire L.I., Pal S., Ironside J.W., Neumann J., Christie S., Joachim C., Esiri M., Evetts S.G., Rolinski M. (2016). Alpha-synuclein RT -Qu IC in the CSF of patients with alpha-synucleinopathies. Ann. Clin. Transl. Neurol..

[236] Shahnawaz M., Tokuda T., Waragai M., Mendez N., Ishii R., Trenkwalder C., Mollenhauer B., Soto C. (2017). Development of a Biochemical Diagnosis of Parkinson Disease by Detection of α-Synuclein Misfolded Aggregates in Cerebrospinal Fluid. JAMA Neurol..

[237] Baldacci F., Daniele S., Piccarducci R., Giampietri L., Pietrobono D., Giorgi F.S., Nicoletti V., Frosini D., Libertini P., Gerfo A.L. (2019). Potential Diagnostic Value of Red Blood Cells α-Synuclein Heteroaggregates in Alzheimer’s Disease. Mol. Neurobiol..

[238] Johnstone R.M., Adam M., Hammond J.R., Orr L., Turbide C. (1987). Vesicle formation during reticulocyte maturation. Association of plasma membrane activities with released vesicles (exosomes). J. Biol. Chem..

[239] Coleman B.M., Hill A.F. (2015). Extracellular vesicles—Their role in the packaging and spread of misfolded proteins associated with neurodegenerative diseases. Semin. Cell Dev. Biol..

[240] Vella L.J., Greenwood D.L., Cappai R., Scheerlinck J.-P.Y., Hill A.F. (2008). Enrichment of prion protein in exosomes derived from ovine cerebral spinal fluid. Veter- Immunol. Immunopathol..

[241] Street J.M., E Barran P., Mackay C.L., Weidt S., Balmforth C., Walsh T.S., Chalmers R.T., Webb D.J., Dear J.W. (2012). Identification and proteomic profiling of exosomes in human cerebrospinal fluid. J. Transl. Med..

[242] Chiasserini D., Van Weering J.R., Piersma S.R., Pham T.V., Malekzadeh A., Teunissen C.E., De Wit H., Jiménez C.R. (2014). Proteomic analysis of cerebrospinal fluid extracellular vesicles: A comprehensive dataset. J. Proteom..

[243] Saman S., Kim W., Raya M., Visnick Y., Miro S., Saman S., Jackson B., McKee A.C., Alvarez V.E., Lee N.C.Y. (2012). Exosome-associated Tau Is Secreted in Tauopathy Models and Is Selectively Phosphorylated in Cerebrospinal Fluid in Early Alzheimer Disease. J. Biol. Chem..

[244] Fiandaca M.S., Kapogiannis D., Mapstone M., Boxer A., Eitan E., Schwartz J.B., Abner E.L., Petersen R.C., Federoff H.J., Miller B.L. (2015). Identification of preclinical Alzheimer’s disease by a profile of pathogenic proteins in neurally derived blood exosomes: A case-control study. Alzheimer’s Dement..

[245] Hart N.J., Koronyo Y., Black K.L., Koronyo-Hamaoui M. (2016). Ocular indicators of Alzheimer’s: Exploring disease in the retina. Acta Neuropathol..

[246] Koronyo-Hamaoui M., Koronyo Y., Ljubimov A.V., Miller C.A., Ko M.K., Black K.L., Schwartz M., Farkas D.L. (2011). Identification of amyloid plaques in retinas from Alzheimer’s patients and noninvasive in vivo optical imaging of retinal plaques in a mouse model. NeuroImage.

[247] Koronyo Y., Salumbides B.C., Black K.L., Koronyo-Hamaoui M. (2012). Alzheimer’s Disease in the Retina: Imaging Retinal Aß Plaques for Early Diagnosis and Therapy Assessment. Neurodegener. Dis..

[248] Koronyo Y., Biggs D., Barron E., Boyer D.S., Pearlman J.A., Au W.J., Kile S.J., Blanco A., Fuchs D.-T., Ashfaq A. (2017). Retinal amyloid pathology and proof-of-concept imaging trial in Alzheimer’s disease. JCI Insight.

[249] Feke G.T., Hyman B.T., Stern R.A., Pasquale L.R. (2015). Retinal blood flow in mild cognitive impairment and Alzheimer’s disease. Alzheimer’s Dement. Diagn. Assess. Dis. Monit..

[250] Alber J., Goldfarb D., Thompson L.I., Arthur E., Hernandez K., Cheng D., DeBuc D.C., Cordeiro F., Provetti-Cunha L., Haan J.D. (2020). Developing retinal biomarkers for the earliest stages of Alzheimer’s disease: What we know, what we don’t, and how to move forward. Alzheimer’s Dement..

[251] Palermo G., Mazzucchi S., Della Vecchia A., Siciliano G., Bonuccelli U., Azuar C., Ceravolo R., Lista S., Hampel H., Baldacci F. (2020). Different Clinical Contexts of Use of Blood Neurofilament Light Chain Protein in the Spectrum of Neurodegenerative Diseases. Mol. Neurobiol..

[252] Al-Chalabi A., Hardiman O., Kiernan M.C., Chiò A., Rix-Brooks B., Berg L.H.V.D. (2016). Amyotrophic lateral sclerosis: Moving towards a new classification system. Lancet Neurol..

[253] Castrillo J.I., Lista S., Hampel H., Ritchie C.W. (2018). Systems biology methods for Alzheimer’s disease research toward molecular signatures, subtypes, and stages and precision medicine: Application in cohort studies and trials. Methods in Molecular Biology.

[254] Geerts H., Dacks P.A., Devanarayan V., Haas M., Khachaturian Z.S., Gordon M.F., Maudsley S., Romero K., Stephenson D., Brain Health Modeling Initiative (BHMI) (2016). Big data to smart data in Alzheimer’s disease: The brain health modeling initiative to foster actionable knowledge. Alzheimer’s Dement..

